# Modeling Chemical Interaction Profiles: II. Molecular Docking, Spectral Data-Activity Relationship, and Structure-Activity Relationship Models for Potent and Weak Inhibitors of Cytochrome P450 CYP3A4 Isozyme

**DOI:** 10.3390/molecules17033407

**Published:** 2012-03-15

**Authors:** Yunfeng Tie, Brooks McPhail, Huixiao Hong, Bruce A. Pearce, Laura K. Schnackenberg, Weigong Ge, Dan A. Buzatu, Jon G. Wilkes, James C. Fuscoe, Weida Tong, Bruce A. Fowler, Richard D. Beger, Eugene Demchuk

**Affiliations:** 1 Division of Toxicology and Environmental Medicine, Agency for Toxic Substances and Disease Registry, Atlanta, GA 30333, USA; Email: YTie@cdc.gov (Y.T.); BMcPhail@cdc.gov (B.M.); BFowler@icfi.com (B.A.F.); 2 Division of Systems Biology, National Center for Toxicological Research, U.S. Food and Drug Administration, Jefferson, AR 72079, USA; Email: Huixiao.Hong@fda.hhs.gov (H.H.); BruceA.Pearce@fda.hhs.gov (B.A.P.); Laura.Schnackenberg@fda.hhs.gov (L.K.S.); Weigong.Ge@fda.hhs.gov (W.G.); Dan.Buzatu@fda.hhs.gov (D.A.B.); Jon.Wilkes@fda.hhs.gov (J.G.W.); James.Fuscoe@fda.hhs.gov (J.C.F.); Weida.Tong@fda.hhs.gov (W.T.); Richard.Beger@fda.hhs.gov (R.D.B.); 3 Department of Basic Pharmaceutical Sciences, West Virginia University, Morgantown, WV 26506-9530, USA

**Keywords:** structure-activity relationship, SAR, QSAR, SDAR, docking, molecular modeling, inhibitor, CYP3A4, drug-drug interaction, drug-chemical interaction, DDI, DDCI

## Abstract

Polypharmacy increasingly has become a topic of public health concern, particularly as the U.S. population ages. Drug labels often contain insufficient information to enable the clinician to safely use multiple drugs. Because many of the drugs are bio-transformed by cytochrome P450 (CYP) enzymes, inhibition of CYP activity has long been associated with potentially adverse health effects. In an attempt to reduce the uncertainty pertaining to CYP-mediated drug-drug/chemical interactions, an interagency collaborative group developed a consensus approach to prioritizing information concerning CYP inhibition. The consensus involved computational molecular docking, spectral data-activity relationship (SDAR), and structure-activity relationship (SAR) models that addressed the clinical potency of CYP inhibition. The models were built upon chemicals that were categorized as either potent or weak inhibitors of the CYP3A4 isozyme. The categorization was carried out using information from clinical trials because currently available *in vitro* high-throughput screening data were not fully representative of the *in vivo* potency of inhibition. During categorization it was found that compounds, which break the Lipinski rule of five by molecular weight, were about twice more likely to be inhibitors of CYP3A4 compared to those, which obey the rule. Similarly, among inhibitors that break the rule, potent inhibitors were 2–3 times more frequent. The molecular docking classification relied on logistic regression, by which the docking scores from different docking algorithms, CYP3A4 three-dimensional structures, and binding sites on them were combined in a unified probabilistic model. The SDAR models employed a multiple linear regression approach applied to binned 1D ^13^C-NMR and 1D ^15^N-NMR spectral descriptors. Structure-based and physical-chemical descriptors were used as the basis for developing SAR models by the decision forest method. Thirty-three potent inhibitors and 88 weak inhibitors of CYP3A4 were used to train the models. Using these models, a synthetic majority rules consensus classifier was implemented, while the confidence of estimation was assigned following the percent agreement strategy. The classifier was applied to a testing set of 120 inhibitors not included in the development of the models. Five compounds of the test set, including known strong inhibitors dalfopristin and tioconazole, were classified as probable potent inhibitors of CYP3A4. Other known strong inhibitors, such as lopinavir, oltipraz, quercetin, raloxifene, and troglitazone, were among 18 compounds classified as plausible potent inhibitors of CYP3A4. The consensus estimation of inhibition potency is expected to aid in the nomination of pharmaceuticals, dietary supplements, environmental pollutants, and occupational and other chemicals for in-depth evaluation of the CYP3A4 inhibitory activity. It may serve also as an estimate of chemical interactions via CYP3A4 metabolic pharmacokinetic pathways occurring through polypharmacy and nutritional and environmental exposures to chemical mixtures.

## 1. Introduction

Life is an open biological system of co-interacting chemicals, *i.e.*, it is a mixture of chemicals that is continuously exposed to ambient chemicals through respiratory, ingestive, and transdermal intake. Although toxicological or pharmacological effects are commonly associated with a single chemical, exposure to a single chemical entity does not take place. Instead, people are exposed to a mixture of drugs, nutrients, and environmental pollutants simultaneously. The magnitude of systemic health effects caused by exposure to a chemical mixture could be additive if the components of the mixture act independently or non-additive if appreciable interactions between at least some of the components take place [1]. When chemical interactions take place, the estimation of joint response from the known responses to individual components of the mixture and their interactions becomes a problem of combinatorial complexity. Dosage of individual components (“dose makes the poison”), duration of exposure, and the intrinsic strength of interactions between the chemicals are some of the factors determining the overall clinical manifestation. Often at low doses, when biochemical pathways are far from saturation, the interactions are additive. Additivity is typical of long-term systemic exposures to environmental pollutants at hazardous waste sites [1,2]. However, the magnitude of the response to an otherwise low-hazard dose or exposure may change dramatically if the dosage of even one component in an exposure mixture is significantly increased (e.g., a medication is administered) [3].

Exacerbated effects of drug/environment interactions are encountered in the rapidly growing subpopulation of elderly because: (1) body functions steadily diminish and physiological sensitivity increases as aging progresses; (2) pre-existing chronic conditions are common and multiple, and they are being treated by chronic administration of multiple drugs; and (3) contaminants that are lipophilic or stored in bone tissue are being released as bone density and the overall fat slowly dissipate with the increasing age of seniors [4]. The released contaminants may result from: (1) past exposures, including occupational; and (2) exposures to persistent pollutants that are now banned, such as lead in gasoline, dichloro-diphenyl-trichloroethane (DDT), polychlorinated biphenyls (PCBs), and polychlorinated dibenzo-dioxins (PCDDs) [5,6].

Due to polypharmacy, drug-drug/chemical interactions (DDCIs) may be responsible for many detrimental health effects, yet at present their unequivocal characterization is difficult during the preclinical evaluation stage of drug development. Because many drugs are metabolized by cytochrome P450 (CYP) enzymes, other concurrently administered drugs, nutrients, and environmental pollutants that bind to these enzymes may alter blood and tissue concentration of the drug and, therefore, increase the potential for adverse health effects [7,8,9,10,11,12,13]; this circumstance is especially important for drugs with narrow therapeutic indices. Such DDCIs can either increase or decrease the metabolic activity of a CYP enzyme, much like genetic polymorphisms alter patient’s enzymatic activity.

As a part of the microsomal mixed-function oxidase (MFO) enzyme system, CYP enzymes comprise a large group of multifunctional heme-thiolate monooxygenases involved in phase I metabolism. The activity of CYP enzymes is regulated at multiple levels. First, there are multiple binding sites on some, if not all, CYPs [14]. Effector molecules bound to these sites may activate or inhibit the catalysis. Competitive, non-competitive, and mixed inhibition of CYP enzymes can occur [15,16]. Next, the CYP catalytic cycle requires a transfer of two electrons that are delivered by the NADPH regeneration system. Components of this system include NADPH-CYP reductase as a primary component, and at least cytochrome *b*_5_ and NADH-cytochrome *b*_5_ reductase [17]. Altering the electron transfer or oxygenation may also affect the CYP activity. The macroscopic rate of catalysis depends on enzyme concentration. Therefore, CYP inducers, blockers, and dependent nuclear factors play an important role in CYP metabolism. The concentration of substrate available for oxidation often depends on the active transport of substrate to endoplasmic reticulum. Induction and inhibition of transporters is another mechanism that controls DDCIs [18]. In addition, other pharmacokinetic factors, dosage, partial overlap of CYP phenotypes, sequential metabolism, and unaccountable or unknown factors may affect the apparent *in vivo* drug inhibition and DDCIs.

The complex picture of apparent (e.g., clinical) CYP inhibition represents a challenge for unambiguous *in vitro*-to-*in vivo* extrapolation [19,20]. Recommendations for “dosing and labeling,” formulated by the U.S. Food and Drug Administration (FDA) in vetted [21] and draft [18] documents, describe distinctions between the clinical pharmacokinetic- (*in vivo*) and *in vitro*-derived information. The pharmacokinetic picture of DDCIs is more complex. Many DDCIs are transient and context dependent, so they only apply under specific time frames, doses, or other environmental or genetic conditions. This information may or may not be on a drug label, especially when the drug is first released on the market and full understanding of its therapeutic mode of action and contraindications is still emerging. Often, it takes years after the drug is released on the market to find out the optimal dosage, contra-indications, or both—a process that may involve multiple passes between the clinic, FDA, and industry, conjugated to iterative revisions of the drug label, before an optimal and safe regimen of drug administration is established [22]. Because DDCIs are dose and time dependent, a DDCI may occur when the putatively interacting substances are administered around the same time, but not when they are administered several hours apart. Similar considerations would apply to the route of administration; recently it was pointed out that “contraindications with intravenous midazolam are inconsistent” [23]. However, “oral midazolam has the most potential for serious reactions. Because of the high first-pass metabolism with oral midazolam, higher doses are often used. When first-pass metabolism is inhibited, the amount of drug reaching the system is much higher” [23]. To help with the initial assessment of putative DDCIs in which new drugs may be involved, the FDA documents also formulate guidance to using *in vitro* data for the purpose of drug labeling [21] (also, see the Experimental Section). We are unaware of statistics about drug labels on which the *in vitro* information is present, but it is known that some DDCIs observed *in vitro* are not clinically significant, while others observed *in vivo* are not captured by *in vitro* methods [24]. The scientific literature rises a concern that DDCI warnings on some drug labels may be ineffective [22,23,25,26,27,28]. Van der Sijs *et al*. [28] have suggested that European drug labels may contain “false positive” alerts (from the clinician point of view, also known as clinician’s “alert fatigue” [29]) and that “these alerts should be further evaluated for possible improvements in specificity, information content or handling efficiency” [28]. According to a meta-analysis study from the same author, clinicians override up to 96% of drug safety alerts [25,26,27]. To tackle the problem, some health care providers develop their own electronic clinical decision support systems (incorporated in the computerized provider order entry systems as a part of electronic medical record systems), while others use DDCI information formulated by proprietary third-party vendors [30,31,32]. To reduce the effect of false-positive alerts on *in silico* modeling, the present work relied on inhibition potency knowledge from similar clinically relevant sources [32,33]. A reliable clinically-relevant *in silico* DDCI system of alerts has the potential to become an effective risk-management alternative compared with *in vitro* testing.

Extrapolation from preclinical results is intricate. Currently, high-throughput screening (HTS) is often used in drug development. The HTS data are usually collected from *in vitro* microsomal bioassays in which: (1) an ersatz MFO system is reconstructed from recombinant components [34]; (2) a chemical derivative of luminescent beetle luciferin (which is converted by CYP to luciferin) is used as a substrate; and (3) libraries of drugs and drug-like compounds, perhaps, synthesized in the process of drug discovery, are tested for activity of CYP inhibition, activation or both. In an HTS experiment, the rate of substrate conversion to products may either increase (activation) or decrease (inhibition), or either remain unaffected or mutually contradictory at multiple concentrations of the tested compound (inconclusive). The activity is usually expressed by a concentration of the tested compound, which changes the rate of reaction at a specified concentration of substrate by 50%. This is an inhibition constant of 50% (IC_50_) if the activity is decreased. By theory (see the Experimental Section), IC_50_ itself is inappropriate for *in vivo* inference; however, it can be related to *in vivo* health effects under certain assumptions if the physiological concentration of the inhibitor (drug or other chemical) in microsomes of the liver is known. Unfortunately, the latter is difficult to appropriately determine prior to clinical trials.

The first and potentially most severe DDCIs observed in the clinic are expected to be caused by strong binders to phase I enzymes involved in drug metabolism. Potentially, any chemical that is metabolized by, or inhibits, CYP P450 enzymes can competitively inhibit the same enzyme metabolism of other drugs or chemicals. CYP3A4 is the most abundant CYP P450 isoform in the human liver, constituting 30–40% of the total amount of spectroscopically detectable CYP P450 enzymes [35,36]; the amount of hepatic CYP3A4 can be even further increased by induction as much as 60% [37]. It is also the dominant CYP isoform in the small intestine, comprising more than 80% on the enteric-CYP pie chart [36]. It biotransforms endobiotics such as steroids, fatty acids, prostaglandins, lipid-soluble vitamins, and many structurally diverse xenobiotics, including drugs, carcinogens, and environmental pollutants [38]. Overall, it is responsible for the metabolism of nearly 50% of known drugs on the market [39]. Among steroids, hepatic CYP3A4 catabolizes testosterone, progesterone [40], and, together with CYP1A2, as much as 80% of estradiol [41,42]. In this way, CYP3A4 contributes to regulation of endocrine homeostasis. Changes in the levels of reproductive hormones and their metabolites are associated with a gamut of physiologic and adverse health effects [43], ranging from changes in physical appearance to cancers [41,42,43,44,45,46]. Inhibition and transcriptional modulation of CYP3A4 activity by drugs and environmental pollutants (such as DDT, PCBs, and PCDDs) may inadvertently affect these processes by changing the levels of steroid hormones and their metabolites [47,48]. The effects of interference of drugs and environmental pollutants with steroid metabolism may be more significant among special populations. For instance, the activity of CYP3A4 is greater among the residents of Danish Faroe Islands who are dietary exposed to high levels of PCBs and other persistent organohalogen pollutants through traditional consumption of pilot whale blubber [5]. Consistent with its importance, extensive modeling efforts have been made to identify compounds that inhibit the CYP3A4 activity [33,49,50,51]. Efforts have been made to estimate the IC_50_ of *in vitro* inhibition of CYP3A4 by chemical compounds using quantitative structure-activity relationship (QSAR) modeling [51,52]. However, we are unaware of attempts to model the *in vivo* strength of inhibition.

Kinetic and crystallographic studies reveal unusually wide substrate adaptability of CYP3A4, which makes it susceptible to inhibition by a wide variety of chemical groups, and, consequently, difficult to model chemical interactions. Chemicals can act as inducers, effectors, substrates, or inhibitors of CYP3A4 [53], which makes accurate modeling even more challenging. Therefore, using information from clinical trials, we developed multiple *in silico* models of chemical binding and inhibition of the CYP3A4 isozyme, which can be used to estimate *in vivo* DDCIs.

Each method targets different features of the chemicals or drugs: Spectral data-activity relationship (SDAR) modeling relates nuclear magnetic resonance (NMR) spectra to its activity [54]; structure-activity relationship (SAR) estimates its activity by sub-structural fragments, physical-chemical properties or both [52]; and molecular docking combined with logistic regression (DLR) targets the intimate details of protein-ligand interactions [55,56]. We also aimed to gain from the consensus ofthese *in silico* models instead of relying on a single method. These models of DDCIs may be useful for regulatory agencies and public health because they provide *a priori* estimates of DDCIs before they are introduced into patients [57].

## 2. Results and Discussion

### 2.1. Comparison of in-Vitro-Bioassay and Clinical Data

With the advances in laboratory robotic technologies, increasing attention has been drawn to CYP HTS data and its use in DDCI model development [52,57,58,59,60,61,62,63,64,65]. FDA recommends using these data at the drug-development stage but the final drug labeling is based on clinical trial data [18]. Therefore, it is of interest to compare the HTS bioassay and clinical inhibition data (since the latter were used for supervised machine learning in the present work).

The data from two HTS bioassays for CYP3A4 inhibition were available in the PubChem^™^ database [66], assay identification numbers (AIDs) 884 [67,68] and 1851 [67,68]. AID 1851 was a combined bioassay with inhibition data for five CYP isoforms, including 3A4; both bioassays covered large chemical libraries. AID 884 tested 14,155 substances for 13,072 compounds [67,68], and AID 1851 tested 17,143 substances for 16,555 compounds [67,68]. However, many tested compounds were not on the list of FDA-approved drugs. Presumably, these non-drugs were compounds rejected at the research and development stages of the drug discovery process. Fewer than 7% of the compounds in AID 1851 have been identified in PubChem^™^ as drugs, and no compounds have been tagged as drugs in AID 884. A query of the bioassays with a 121-compound dataset of the present work revealed that only 46 and 34 of them were present in the libraries of AIDs 884 and 1851, respectively. Extrapolation from these numbers (7/100 × 16555 × 46/34/13072) suggests that about 12% of drugs may be present in AID 884, assuming equal ratios of the overlap between our database and the subsets of drugs in each of the bioassays. Consequently, FDA-approved drugs were poorly represented in each of the bioassays.

A tested outcome of the AID 1851 bioassay was “whether a compound inhibited pro-luciferin conversion with any of the five isozymes” [67]. Our reanalysis of the raw kinetic data recorded in PubChem^™^ for the aforementioned 34 chemicals did not provide additional information (as compared with AID 884). Therefore, only the outcomes of AID 884 were used for the comparative analysis presented in Table 1. In AID 884, the dose-response curve has been constructed using a luciferin-labeled CYP3A4 substrate (6'-phenylpiperazinylyl) [68]. For most chemicals, the dose-response curve has been measured only once. Multiple curves were only present for chemicals obtained from more than one vendor. For these chemicals, a mean IC_50_ and its 95% confidence interval (CI) were calculated. For single-curve chemicals, a single-point IC_50_ value (as given by the dose-response curve) and a mean CI, averaged over all multiple-curve CIs, were applied.

**Table 1:** Extrapolation from *in vitro* bioassay to *in vivo* pharmacokinetic CYP3A4 inhibition data, and data categorization.

Name	Clinical *Category	IC_50_ (95% CI),AID #884 (µM)	MRDD	BA (%)	*R*-1 (95% CI)	C-1	BA-ADME	*R*-2 (95%CI)	C-2	*R*-3 (95% CI)	C-3	Cmax (µM)	*R*-4 (95%CI)	C-4
clotrimazole	P	0.07 (0.00,0.17)	6.67 ^‡^ [69]	100	280.33 (115.25,∞)	P	30–70	196.53 (80.98,∞)	P	280.33 (115.25,∞)	P	0.087 [70]	2.26 (1.51,∞)	ND
isoniazid	P	9.92 (0.00,26.37)	15 [71]	80 [72,73]	12.03 (5.15,∞)	P	>70	12.03 (5.15,∞)	P	12.03 (5.15,∞)	P	76.61 [70]	8.73 (3.91,∞)	P
diltiazem	P	3.98 (0.00,10.88) ^†^	8 [74]	38 [72,73]	2.84 (1.67,∞)	ND	<30	2.45 (1.53,∞)	ND	5.85 (2.77,∞)	P	0.356 [70]	1.09 (1.03,∞)	ND
ketoconazole	P	0.13 (0.00,7.03) ^†^	8 [75]	50 [76]	60.79 (2.07,∞)	P	30–70	84.70 (2.50,∞)	P	120.58 (3.14,∞)	P	7 [77]	56.60 (2.00,∞)	P
glibenclamide	W	5.99 (0.00,12.80)	0.29 [78]	80 [72,73]	1.08 (1.04,∞)	ND	30–70	1.07 (1.03,∞)	ND	1.10 (1.05,∞)	ND	0.2 [77]	1.03 (1.02,∞)	ND
methoxsalen	W	10.50 (3.39,17.61)	0.6 [74]	26 [79]	1.07 (1.04,1.21)	W	>70	1.26 (1.16,1.82)	ND	1.26 (1.16,1.82)	ND	0.184 [80]	1.02 (1.01,1.05)	ND
omeprazole	W	10.00 (3.10,16.9) ^†^	2 [74]	47 [72,73]	1.27 (1.16,1.88)	W	30–70	1.41 (1.25,2.31)	W	1.58 (1.34,2.87)	W			
clemastine	W	5.01 (0.00,13.48)	0.134 [81]	37 [72,73]	1.03 (1.01,∞)	ND	30–70	1.05 (1.02,∞)	ND	1.08 (1.03,∞)	ND	0.00233 [82]	1.00 (1.00,∞)	ND
dexmedetomidine	W	0.16 (0.00,7.06) ^†^	0.2 ^‡^ [83]	100	7.30 (1.14,∞)	ND	30–70	5.71 (1.10,∞)	ND	7.30 (1.14,∞)	ND			
lansoprazole	W	20.48 (7.63,33.33)	0.5 [74]	81 [72,73]	1.05 (1.03,1.14)	ND	30–70	1.05 (1.03,1.12)	ND	1.07 (1.04,1.18)	ND	1.92 [84]	1.09 (1.06,1.25)	ND
nifedipine	W	11.29 (7.70,14.88)	1.71 [85]	50 [72,73]	1.22 (1.17,1.32)	ND	30–70	1.31 (1.23,1.45)	ND	1.44 (1.33,1.64)	W	0.027 [86]	1.00 (1.00,1.00)	ND
pilocarpine	W	15.85 (8.95,22.75) ^†^	0.5 [74]				<30	1.05 (1.03,1.08)	ND	1.15 (1.11,1.27)	ND	0.099 [70]	1.01 (1.00,1.01)	ND
mitoxantrone	W	25.12 (18.22,32.02) ^†^	2 ^‡^ [87]	100	1.18 (1.14,1.25)	ND	<30	1.05 (1.04,1.07)	ND	1.18 (1.14,1.25)	ND	0.36 [70]	1.01 (1.01,1.02)	ND
irbesartan	W	15.85 (8.95,22.75) ^†^	5 [74]	70 [72,73]	1.52 (1.36,1.91)	W	30–70	1.52 (1.36,1.91)	W	1.74 (1.51,2.30)	W	7.98 [88]	1.50 (1.35,1.89)	W
losartan	W	19.95 (13.05,26.85) ^†^	1.67 [74]	36 [72,73]	1.07 (1.05,1.11)	ND	30–70	1.14 (1.10,1.21)	ND	1.20 (1.15,1.30)	ND	0.596 [89]	1.03 (1.02,1.05)	ND
sildenafil	W	10.00 (3.10,16.9) ^†^	1.67 [74]	38 [72,73]	1.13 (1.08,1.43)	ND	30–70	1.25 (1.15,1.79)	ND	1.35 (1.21,2.14)	ND	0.447 [90]	1.04 (1.03,1.14)	ND
pergolide	W	12.59 (5.69,19.49) ^†^	0.05 [74]	38 [72,73]	1.00 (1.00,1.01)	ND	<30	1.00 (1.00,1.01)	ND	1.01 (1.01,1.03)	ND			

* P = potent inhibitor, W = weak inhibitor; ^†^ only a single measurement was available for this chemical, therefore an average error bar for multiple measurements of ±6.9 µM was applied; MRDD: Maximum recommended daily dose in units of mg/kg/day per oral administration, except when labeled with ^‡^, which denotes intravenous administration; BA: bioavailability; BA-ADME: BA calculated using the Human Oral Bioavailability module of the ACD/ADME^™^ suite, and then the greatest BA in the range was used for calculating *R*; *C*_max_: *in vivo* peak plasma concentration of inhibitor; *R*: *in vivo* AUC fold change; *R*-1: *R* extrapolated from the reference MRDD and BA using Equation 6; *R*-2: *R* calculated using BA-ADME; *R*-3: *R* calculated assuming BA of 100%; R-4: *R* extrapolated from *C*_max_; C-1, -2, -3, -4: Inhibitor categorization based on *R*-1, -2, -3, -4, respectively.

As shown in Table 1, CIs on the IC_50_ values were large. A 95% CI exceeded the mean for 71% of the 17 analyzed drugs. For inhibitors with low IC_50_ values, which included the potent inhibitors, the statistics was even poorer: A CI exceeded the mean for 100% of inhibitors with IC_50_ < 10 µM. Obviously, the power of analyzed HTS data was hardly sufficient for making inferences, which highlights the importance of study design in HTS experiments and suggests that a single-point cut-off on IC_50_ [58,91] is inappropriate for categorizing HTS data.

Two kinds of changes in activity were observed in the HTS bioassay: Compounds with positive activity readings, which increased the rate of catalysis of the test substrate, have been categorized as activators; compounds with negative activity readings have been categorized as inhibitors. The dose-response curve has been interpreted in PubChem^™^ for 19 of the 46 drugs of the training set covered by AID 884, while the kinetics results for 27 drugs have been inconclusive. Of the 19 drugs, 17 were inhibitors and two were activators. These numbers suggest an approximate 10% mismatch between the clinical and HTS inhibition data (because all drugs recruited in the present study were categorized as inhibitors based on clinical reports).

*In vitro* IC_50_ values have been reported for many compounds in the AID 884 bioassay, but their relation to *in vivo* inhibition has not been determined. To map the *in vitro* data to clinically observable *in vivo* bioactivity, the IC_50_ values were extrapolated to pharmacokinetically relevant quantities. Following the FDA guidance [18], a ratio, *R*, of the inhibited area-under-the-curve (AUC) to uninhibited AUC was used to quantify the *in vivo* inhibition activity. The extrapolation was carried out using Equation 6 provided in the Experimental Section. Two approaches were conducted to estimate the physiological concentration of the inhibitor: One by the maximum recommended daily dose (MRDD) and bioavailability, and another by the greatest reported clinical plasma peak concentration, *C*_max_, which was taken as the maximum hepatic inlet concentration. The results of calculations and subsequent categorization are presented in Table 1. A threshold of 2 on *R* was applied for inhibition strength categorization. Drugs with *R* > 2 were categorized as potent inhibitors (P = “strong” ∪ “moderate”), while those with 1.25 ≤ *R* ≤ 2 as weak inhibitors (W). Compounds with *R* lower than a threshold of 1.25 were considered to be irrelevant to clinical manifestations of CYP3A4 inhibition.

An agreement between the *in vivo* and *in vitro* categorization was poor. More than half of the *in vitro* inhibitors could not be categorized. The categorization was not feasible either because the CI was crossing a threshold on *R* or because the calculated *R* was outside the range of *in vivo* inhibition. When reported bioavailability values were used for calculating *R*-1, three of four potent inhibitors and three of 12 weak inhibitors were identified correctly by *in vitro* data, giving a 37.5% rate of success.

Calculations of *R* with bioavailability derived using ACD/ADME^™^ and with the default bio-availability of 100% were carried out to test a hypothesis about their utility in the calculations of *R*. Bioavailability is a challenging topic for both *ab initio* modeling and cross-chemical extrapolation [72,92]. Hypothetically, a bioavailability surrogate could be applied to non-drugs in HTS libraries and other compounds, for which bioavailability has not yet been determined. When using the ACD-calculated bioavailability or a default bioavailability of 100%, the results of *R* calculations were similar to aforementioned *R*-1. With the ACD-calculated bioavailability, three of four potent inhibitors and two of 13 weak inhibitors were correctly attributed, giving a 29% success rate; with the default bioavailability, four of four potent inhibitors and three of 13 weak inhibitors were correctly attributed, giving a 41% success rate. Thus, based upon the results of the present study, no evidence suggested that the default bioavailability of 100% in calculations of *R* was not appropriate.

Given a well-recognized uncertainty associated with experimentally determined MRDD and bioavailability values, directly measured plasma *C*_max_ values may be thought of as an appealing alternative. When *C*_max_ values compiled from the literature and clinical trials (Dr. Minjun Chen personal communication) were attempted in *R*-4 calculations, only two of four potent and one of 10 weak inhibitors were assigned correctly. This translates into a 21% success rate, which is almost twice as poor as the success rate of *R*-3 calculations with reported MRDD and default bioavailability values.

Within the groups of inhibitors, which were successfully categorized using the *in vitro* data (non-NDs in Table 1), no gross miscategorization upon *in vitro* to *in vivo* extrapolation was observed, although an overlap between the clinical dataset and PubChem^™^ data was small. However, little of the PubChem^™^ data were useful for potency categorization because of the low power of underlying IC_50_ values. Similarly, the IC_50_ values alone, without extrapolation to *in vivo*, were of little utility for inhibitor categorization. Perhaps, deficiencies in the *in vitro* data can be eliminated in the future with improved study design, after which the HTS information may become appropriate for modeling DDCIs. Still, about a 10% mismatch between the HTS measurements and results of clinical trials may be anticipated. This number is relatively small provided the complexity of the *in vivo* responses; the latter is especially important for CYP3A4. CYP3A4 regulation is perhaps the most complex of all CYP isoforms, because of both peculiarities in the structure/kinetics and system-biological effects. For instance, both hepatic and enteric forms of CYP3A4 are expressed in large relative amounts, and it has been shown that these forms are kinetically distinct [93] and may contribute differently to the pharmacokinetic profile of a xenobiotic. Also, the activity of CYP3A4 is regulated at multiple levels by inducers, transporters, and other factors [53]. Altogether, a 10% mismatch does not seem unreasonable but suggests that, even in theory, the performance of HTS data in dissecting DDCIs is capped below 100% because of causal oversimplification by an *in vitro* system. To the contrary, *in vivo* clinical data are exempt of such a limitation, while their utility depends on a technical implementation of supervised machine learning; hypothetically, an advanced machine classifier based on clinical data can be fully successful. However, *in vitro* systems enable the study of more combinations of agents than the clinical approaches do. Perhaps, future computational approaches bearing on both strategies will become most effective.

The PubChem AID 884 bioassay was also used to examine an association between the molecular weight (MW) and inhibition properties of chemical compounds. A histogram of MWs of compounds tested for CYP3A4 activity in the AID 884 bioassay was unimodal. It followed a skewed bell-shape distribution with the mode at 330 ± 30, median at 331 Da, mean at 344 Da, and geometric mean at 316 Da. There were 3,680 compounds active in the test for CYP3A4 inhibition, and 7,418 were inactive. The histograms of active and inactive compounds were also unimodal and similarly shaped, but the descriptive statistics were different. The mode, median, mean, and geometric mean of inactive compounds were 285 ± 20, 294, 311, and 280 Da. Those of active compounds were 350 ± 10, 373, 397, and 378 Da. The 95% parametric CIs on the means were less than 5 Da. These data suggested that inactive and active compounds dominated different bands on the MW range: The low- and high-MW bands were populated with inactive and active compounds, respectively.

The MW histograms of active and inactive compounds were much overlapped and skewed. Because of that, the optimal point for MW banding was not immediately clear. Ostensibly, the point could be determined if the underlying theoretical distributions of the active and inactive compounds would have been known. However, the histograms were imperfectly shaped. An expectation was that MW of a randomly drawn organic compound follows the lognormal distribution. Descriptive statistics of the lognormal distribution satisfy the following inequality: {mode = *e*^(^^μ − ^^σ^2)^} < {median = *e*^μ^} < {mean = *e*^[^^μ + (^^σ^2)/2]^}, where μ and σ^2^ are the mean = median = mode and variance of the reciprocal normal distribution on the logarithmic scale. Only the descriptive statistics of active compounds satisfied the inequality. The MW of active compounds was approximately lognormally distributed with an estimated mean of 2.578 and standard deviation (SD) of 0.1313 on the log_10_ scale. For inactive compounds and dominated by them the full library, the inequality did not hold. It means that although the general tendency in these histograms was lognormal (as indicated by the median < mean), the lognormal distribution was inappropriate because the theoretical and observed bell shapes did not match. Moreover, the histograms of inactive compounds and of the full library could not be meaningfully fit with neither of normal, lognormal, or Weibull distributions. In these histograms, the low-MW side of the bell shape seemingly followed the normal distribution, while the high-MW side resembled a lognormal density. Therefore, unlikely the library of AID 884 compounds was a balanced random draw. The structure of the distribution suggested an unidentified bias in the HTS dataset that was caused, perhaps, either by different vendors of the tested substances, or different subsets of the substances, or maybe several biased draws from the chemical space. Conceivably, the poor-fit histograms could be described by a superposition of several random processes, but such level of details was outside the scope of the present study. Therefore, an alternative approach was adopted, in which a threshold for MW banding was empirically extracted directly from the histograms.

**Figure 1:**
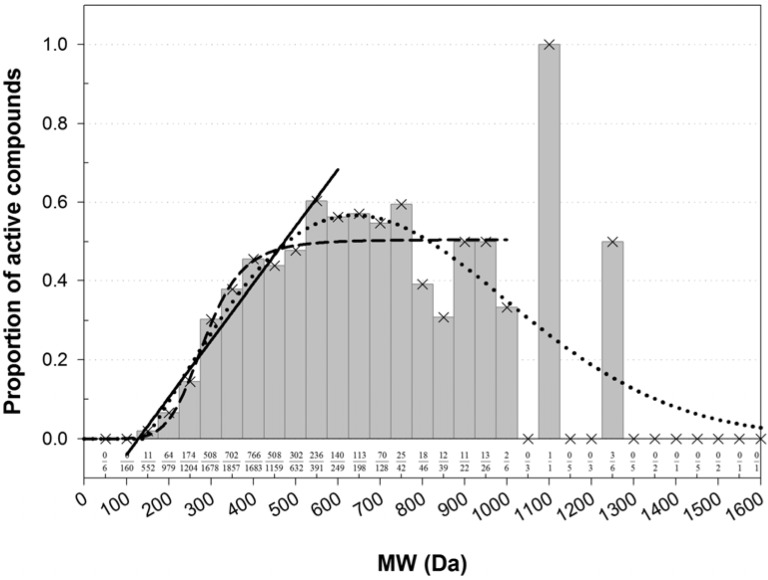
Normalized histogram of MW of compounds active in AID 884. The number of active compounds in each bin was normalized by the total number of active and inactive compounds in that bin. The proportions of active compounds are labeled at the bottom. The histogram shape was fitted with functions from the bell-shaped and sigmoid families and with a linear function. In the each family, only the best fit was retained. A fit with a four-parameter Weibull function (as implemented in the SigmaPlot^®^ software; a = 0.5670, b = 686.7269, c = 2.0494, x_0_ = 639.5385) was carried out on the entire domain of MW, *r*^2^ = 0.5837. A fit with the logistic function (*y* = 0.5047/[1 + (285.1/x)6.266]) was carried out for MW ≤ 950 Da, *r*^2^ = 0.8970. A line (*y* = 0.001437*x* − 0.1825) was fitted to the bins in the range 150–550 Da, *r*^2^ = 0.9479. The fitted shapes intersected at about the same point of 450 Da, which was taken as a threshold separating the MW range in a low-MW band that was depleted with active compounds and a high-MW band that was enriched with active compounds.

Instead of a ratio of two theoretical distributions, the enrichment with active compounds was studied using a normalized histogram (Figure 1). Clearly, there was an association between MW and the frequency of active compounds. The bins were most populous in the low-MW range. There, a fraction of active compounds increased in a quasi-linear fashion from zero at about 60–172 Da to a quasi-plateau that began at 450–550 Da (Figure 1). Poor statistics above 1,000 Da prevented confident characterization of the fraction of active compounds at high MWs. However, there was no evidence that the fraction was increasing with increasing MW. In the high-MW band the fraction was either constant at about 0.5, or maybe decreasing at very high MWs. However, in the area of high counts, the normalized histogram could be almost equally-well fitted with a linear, sigmoid, or bell-shaped dependence (Figure 1). The shapes of different kind intersected at about 450 Da, which was taken as a threshold for MW banding. 

Among the active compounds, 2,733 had MW lesser and 947 greater than the threshold. The respective proportions of active/(active + inactive) were *ν*_1_ = 2733/9278 = 0.2946 (95% CI: 0.2853–0.3039) and *ν*_2_ = 947/1820 = 0.5203 (95% CI: 0.4974–0.5433). One-tailed Z-test for two proportions suggested that these proportions were statistically significantly distinct; there was an almost 100% chance (*p* < 2.235 × 10^−78^) that the proportion of active compounds in the low-MW band was less than the proportion of active compounds in the high-MW band. This was in agreement with Lipinski's rule of five, which MW condition postulates that MW of drugs with favorable ADMET properties shall not exceed 500 Da [94]. The ratio of proportions was *ν*_2_/*ν*_1_ = 1.766 (95% CI: 1.670–1.862), *i.e.*, it was about twice more likely to encounter an inhibitor of CYP3A4 among compounds in the high-MW band compared to the low-MW band.

### 2.2. Docking Classifier of Potent and Weak Inhibitors of CYP3A4

Crystal structures of human CYP3A4 with and without inhibitor correlated with the well-known structural flexibility of the protein. The flexibility and consequent substrate adaptability of CYP3A4 observed in the structures were consistent with atypical kinetic behavior that has been described for CYP3A4 [95,96,97]. MW of CYP3A4 inhibitors studied crystallographically ranged from 226 to 734 Da, while the volume of active site [98], conjugated to the size of substrate, increased up to 80%. Multiple substrate binding modes (productive and non-productive) as well as multiple binding sites (at the site entrance, the catalytic site, and even the secondary binding site) were observed in the crystallographic complexes. Based on the structural analysis, four protein data bank (PDB) structures with seven possible binding pockets were selected for docking studies (refer to the Experimental Section for details). Four molecular docking programs were applied and, thus, 28 sets of docking scores were generated.

The docking scores were combined using a logistic regression (LR) procedure. LR variables were selected either by using the forward-stepwise, backward-stepwise, or a manual selection procedure. Although the stepwise variable selection procedures are widely used, they are not perfect, and their drawbacks are well understood [99]. Therefore, an emphasis in the present work was made on manual selection (a so-called “full” model [99]).

**Figure 2:**
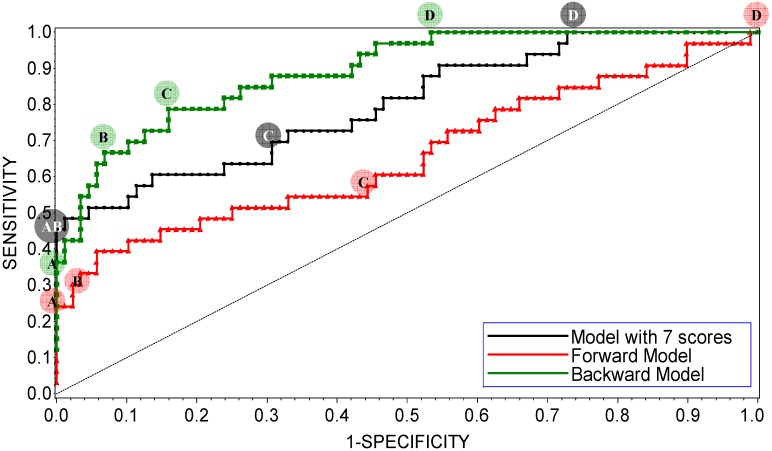
ROC plot of the interim DLR models for potent and weak inhibitors of CYP3A4. A, B, C, and D denote maximum specificity, maximum correct classification, equal sensitivity and specificity, and maximum sensitivity points on the ROC curves, respectively.

The LR models were trained using 33 potent and 88 weak CYP3A4 inhibitors (see the Experimental Section for details). Receiver operating characteristic (ROC) curves of the refined interim models are shown in Figure 2. ROC curves were calculated by varying the cutoff probability point from one on the left side to zero on the right side of the ROC plot; which cutoff is theoretically most appropriate is a matter of debate. In practice, the choice of cutoff is often driven by the utility or by cost-effectiveness considerations. Common choices are 0.5 (SAS^®^ default), or a cutoff point based on prior or posterior probabilities. The prior or posterior cutoff points are appealing when categories are highly unbalanced, that is when events in one of the categories are very rare. In this case, a rare-events correction to the model intercept can be used [100]. In the present work, the categories of the training set were split approximately 1:3, which suggests that the minority type was unbalanced but not rare. In this case, increasing the size of the training set and using the default cutoff of 0.5 is a preferred way of handling the data; appropriately increased size of the training set allows for the adequate representation of patterns that comprise the minority distribution.

As shown in Figure 2, maximum correct classification and equal sensitivity/specificity could not be achieved with the same cutoff point. A balanced model with equal sensitivity and specificity could be obtained using a cutoff based on the prior probability of categorization of potent inhibitors (33/121), which comprised the minority population (Table 2). However, because the training set was unbalanced, the maximum correct classification could not be achieved with the prior cutoff. The maximum correct classification was achieved with the default cutoff of 0.5. To maximize the correct classification, a cutoff of 0.5 was adopted in the present study.

**Table 2:** Effect of LR probability cutoff on model performance.

Model	Cutoff point	Probability cutoff	Sensitivity	Specificity	Correct classification
Forward model	maximum specificity	0.60	0.15	1.00	0.77
	maximum correct classification	0.50	0.36	0.94	0.79
	specificity = sensitivity	0.24	0.55	0.57	0.56
	maximum sensitivity	0.04	1.00	0.00	0.27
Backward model	maximum specificity	0.80	0.36	1.00	0.83
	maximum correct classification	0.50	0.64	0.94	0.86
	specificity = sensitivity	0.28	0.79	0.78	0.79
	maximum sensitivity	0.08	1.00	0.73	0.47
Manual model	maximum specificity	0.50	0.42	1.00	0.84
	maximum correct classification				
	specificity = sensitivity	0.26	0.68	0.69	0.69
	maximum sensitivity	0.10	1.00	0.27	0.47
Manual model, MW > 450	maximum specificity	0.50	0.72	1.00	0.87
	maximum correct classification				
	specificity = sensitivity	0.43	0.78	0.76	0.77
	maximum sensitivity	0.07	1.00	0.38	0.64

ROC AUC is often used as an empirical statistic for model comparison (and its close relationship to rank tests in non-parametric statistics can be shown). The ROC AUC is interpreted as a measure of model quality. Clearly, if ROC AUC is 1, the model is perfect, and if it is 0.5 (the diagonal), the model is worthless. The calculated ROC AUCs were 0.66, 0.89, and 0.80 for the forward-stepwise, backward-stepwise, and manual selection LR models, respectively. The results suggest that the backward-stepwise and manual selection models were not excellent (ROC AUC > 0.9), however, but they were good (ROC AUC > 0.8).

The AUCs were roughly proportional to the number of docking scores recruited in the model: There were 3, 13, and 7 scores in the forward-stepwise, backward-stepwise, and manual selection LR models, respectively. As can be seen in Figure 2, the backward-stepwise and manual selection models were opposite in terms of specificity and sensitivity, *i.e.*, the backward-stepwise model was better at sensitivity and the manual selection model was better at specificity. Namely, adding more parameters to the model improved representation of the major category of weak inhibitors but it did not help the machine classifier to better learn about the minor category of potent inhibitors, perhaps, because of under population of the latter. There were 121 compounds in the training set. Applying a 1:20 rule for candidate predictor variables in a LR model [101], it was perceived that the backward-stepwise model may be over-trained, while the forward-stepwise may be under-trained. A conservative choice was made in favor of the manually selected “full” model with a balanced number of parameters. The manual selection model showed 100% specificity at the cutoff point of maximum correct classification of the model on the ROC curve (Figure 2), which was achieved using the default categorizing probability cutoff. Thus, the manual selection model with a categorizing probability cutoff of 0.5 was adopted as the final model at the LR parameter-optimization stage.

Docking scores included in the final model are presented in Table 3. The table also shows the contribution and significance of each score. A docking score that was calculated using Surflex^™^ at the 1W0F-catalytic site gave the highest contribution, while the score that was calculated using FRED^™^ at the 2V0M-catalytic site was the next highest contribution. All docking scores were significant for the final model with *p*-values of less than 0.05, except for the 1W0F-entrance site score from FRED^™^, with a *p*-value less than 0.1. Six of seven selected scores were pertinent to the catalytic site. This circumstance suggests that competitive inhibition at the active site is perhaps the major determinant of the high potency of CYP3A4 inhibition, as given by the chemicals of the training set. Thus, allosteric sites, which often result in atypical Michaelis-Menten kinetics, must be those that contribute mostly to weak inhibition of CYP3A4.

**Table 3:** Summary of the final DLR model.

Analysis of Maximum Likelihood Estimates					
Parameter	DF	Estimated Coefficient	Standard Error	Wald χ^2^	Pr > χ^2^
1W0F-catalytic site-by Surflex^™^	1	5.5653	1.5783	12.4342	0.0004
2V0M-catalytic site-by FRED^™^ *	1	−2.2601	0.8146	7.6972	0.0055
2J0D-catalytic site-by Surflex^™^	1	−1.7176	0.6328	7.3684	0.0066
2V0M-catalytic site-by Glide^™^	1	1.5078	0.5545	7.3927	0.0065
2V0M-full active site-by FRED^™^^†^	1	1.4618	0.5385	7.3740	0.0066
1W0F-catalytic site-by Glide^™^	1	−1.2597	0.5007	6.3288	0.0119
1W0F-entrance site-by FRED^™^	1	−1.0350	0.6189	2.7967	0.0845
Intercept	1	−1.8088	0.3556	25.8789	<0.0001

* The “catalytic site” of 2V0M is referred to the ketoconazole molecule in the active site, which has its imidazole nitrogen atom within the bonding distance of the heme iron atom; ^†^ The “full active site” of 2V0M also includes a binding site for the second ketoconazole molecule bound to 2V0M in a non-productive orientation.

The final DLR model constructed using the full training set (the joint model) had a 100% specificity, 42.4% sensitivity, and a rate-of-correct-classification of 84.3%. A tenfold cross-validation (CV) was carried out by splitting the training set in a proportion of 1:9 and leaving out the 10% of the sample. The average model rates-of-correct-classification for ten random splits of the training subsets (*i.e.*, each subset contained 90% of the total sample) and the CV subsets (10% of the total sample in each subset) were 83.8% and 79.3%, respectively.

As shown in the previous section, MW was an important factor affecting inhibition properties of chemical compounds. Similarly to compounds active in the HTS assay, a histogram of MWs of the training-set inhibitors also followed the lognormal distribution (*p* = 0.217, the Shapiro-Wilk test for normality). The mean was estimated at log_10_(381), which was indistinguishable from the HTS geometric mean, and the estimated SD was 0.1885.

**Figure 3:**
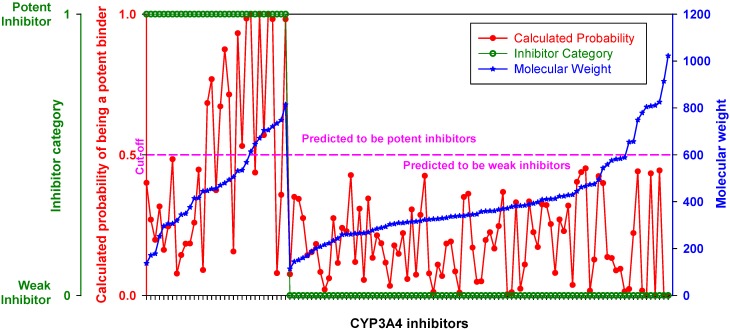
Performance of the DLR model. Potent and weak CYP3A4 inhibitors were sorted by molecular weight, which is shown in blue; their clinical categorization is shown in green; calculated probability scores are depicted in red. The dashed line denotes a chosen probability cut-off for the LR model.

When inhibitors of the training set were ranked by MW, the DLR model showed a stepwise increase in sensitivity at the threshold of 450 Da described in the previous section (Figure 3, Table 2). Below the threshold, the DLR model recognized only one potent inhibitor (tetracycline), whereas above the threshold the model recognized almost 75% of potent inhibitors. Nonetheless, it correctly classified all weak inhibitors, regardless of MW, *i.e.*, there was no false positive error.

Adding MW to the pool of docking scores of LR as the 29th independent variable was not helpful: The coefficient at MW was not statistically significant in the 29- and 7-parameter models (*p*-value of 0.1375 and 0.2217, respectively). The forward- and backward-stepwise procedures also did not select MW as a LR variable. MW was not helpful, perhaps, because it was 35–75% correlated with the docking scores, *i.e.*, it carried redundant information.

To determine if the training set was enriched with potent inhibitors in the high-MW band, a statistical test was carried out. Among 82 inhibitors in the low-MW band, only 15 were categorized as potent; among 39 inhibitors in the high-MW band, the number of potent inhibitors was 18. The respective proportions were *ν*_1_ = 15/82 = 0.1829 (95% CI: 0.1141–0.2801) and *ν*_2_ = 18/39 = 0.4615 (95% CI: 0.3157–0.6143). According to the one-tailed *Z*-test for two proportions, there was a greater than 99.9% chance that the proportion of potent inhibitors in the low-MW band was less than the proportion of potent inhibitors in the high-MW band, *i.e.*, that H_0_: *ν*_1_ − *ν*_2_ = 0 could be rejected in favor of H_1_: *ν*_1_ − *ν*_2_ < 0 (*p* < 6.50 × 10^−4^). The power of the test was 93% (β = 0.0676 at α = 0.05). It meant that the examined inhibitor data were such that the chance of committing a type II error, when accepting the null hypothesis, was almost as low as the targeted chance of type I error (*i.e.*, 5%). Commonly, a study design with the power of 80% is considered acceptable (which implies a four times lesser penalty for a type II error as compared to a type I error) [102]. Thus, chances of committing both type I and type II errors were small, which suggests that the size of the training set was sufficient to confidently conclude that the compounds in the high-MW band were enriched with potent inhibitors of the CYP3A4 isozyme.

The enrichment of the high-MW band with potent inhibitors was about 50%, *i.e.*, it was similar to the enrichment with general CYP3A4 inhibitors described in the previous section. In the low-MW band, however, the fraction of potent inhibitors did not exceed 20%, which was approximately by 30% less than that described for general CYP3A4 inhibitors in the previous section. Combined, these data suggested that in the high-MW band CYP3A4 inhibitors were 2–3 times more likely to be potent compared to inhibitors in the low-MW band. Thus, the effect of MW on potency of CYP3A4 inhibition was more profound than on the category of inhibition itself described in the previous section. Profusion of inhibitors and potent inhibitors in the high-MW band may be related to a greater probability of finding a chiral center in large organic compounds. Because CYP3A4 is stereo- and regio-specific, involvement of enantiomeric decoys unlikely improves but hinders the rate of metabolic reactions. Preliminary examination of inhibitors of other CYP isozymes (data not shown) suggested that (1) a similar dependence between MW and inhibition potency can be observed for inhibitors of other isozymes with adaptive active site, such as 2C9, but unlikely of rigid isozymes, such as 2D6; and that (2) the MW threshold may be unique for each adaptable isozyme. It is not clear at this point, if the MW effect is determined by the volume of the active site, or the active-site adaptability, or the number of binding pockets at the active site (see also Section 2.5). More crystallographic complexes of CYP isozymes and ligands resolved in the future may help to clarify the topic. Also, if the observed phenomenon is indeed related to the probability of a chiral center in the ligand, perhaps, the MW effect will best manifest in isozymes, which spectrum of specificity includes large compounds, *i.e.*, such CYP isozymes as 3A4 and 2C9.

Now, since the actual proportions of potent inhibitors and their respective CIs were known, they could be applied to examine the results of machine classification. Suppose that the number of potent inhibitors in the training set has been forgotten; how many of them will be expected after application of a machine classifier? The prior suggested an expected range for the number of potent inhibitors. If the number of machine-classified potent inhibitors would be within 9–23 in the low-MW band and 12–24 in the high-MW band, that would not contradict a statistical expectation at the 95% confidence level. Similarly, for the whole training set the proportion of potent inhibitors was 0.2727 (95% CI: 0.2013–0.3582). Thus, from 24 to 43 machine-classified potent inhibitors would be expected.

The DLR model correctly classified only 14 potent inhibitors among the 121 compounds of the training set. That was less than categorized 33 potent inhibitors at 99.9% probability (*p* < 1.01 × 10^−3^). The underestimation stemmed from the low-MW band, in which the correct classification (1/82) was less than expected (15/82) at more than 99.99% probability (*p* < 1.15 × 10^−4^). In the high-MW band, however, the null hypothesis (that the proportion of correctly classified potent inhibitors, 13/39, is the same as the proportion of categorized ones, 18/39) could not be rejected (*p* > 0.123, 87.6%). Thus, the natural bias in the number of potent inhibitors categorized across the MW range was exaggerated in the classification results of the DLR model. Perhaps, it was caused either by a bias in model training towards weak inhibitors, which constituted the dominant major category in the low-MW band in a proportion greater than 4:5, or a statistical fluctuation in the data. Taking into account the results of DLR model application to the testing set (see Section 2.6), the latter appeared as a quite likely possibility.

As evident from Figure 3, lowering the probability cutoff point was not helpful, because the domain of probability scores of weak inhibitors almost exactly overlapped with the domain of potent inhibitors in the low-MW band. At 450 Da, the probability scores of potent inhibitors displayed a stepwise increase, which was appropriate for correct classification. The phenomenon could be related either to an almost 1:1 balance between potent and weak inhibitors in the high-MW band, or to an increase in the probability of a chiral center discussed above, or, likely, to the number of contacts that ligand makes at the docking site, *i.e.*, ultimately with the large size and adaptability of the active site pocket on CYP3A4 (see Section 2.5 and Section 3). Another reason could be that the docking study did not cover all binding sites, especially those with a preference for small molecules such as extra-allosteric sites. It is also possible that an effector molecule(s) binding may take place when the substrate/inhibitor is small. For instance, two ketoconazole molecules are bound in one of the crystal structures of CY3A4 used for docking in the present study [98]. However, at least four molecules of 7-benzyloxyquinoline, which has MW less than half that of ketoconazole, bind simultaneously to CYP3A4 [14]. Moreover, unlike other CYP isozymes, the active site pocket of CYP3A4 is enriched with water molecules; the mean coordination number of water molecules in proximity of the heme is at least two to three times greater than in other CYP isozymes [103]. However, the docking procedures employed in the present study did not imply the use of explicit water molecules in docking simulations (although, the mean-field solvent effects were implicitly accounted for in several of the scoring functions). Water molecules in the active site may play an important role in protein-ligand binding. On the one hand, shielding the transition state from water (and thus reducing the activation energy barrier of the transition state) is the main purpose of enzymatic catalysis (which can be achieved either by elasticity of the active site, or by presence in the active site of hetero- or homo-effector molecule(s), similar to the 2V0M structure); but on the other hand, water molecules at high-occupancy hydration sites can mediate the hydrogen-bond network of the active site and stabilize the protein-ligand complex (along with the conformation of the protein and the transition state). Also, they contribute to the free energy of binding. Indeed, the process of ligand binding is also a process of dislodging water from the active site. As water molecules are released from the active site, the entropy of the protein-ligand complex usually goes down, while the entropy of the full system (which also includes the solvent) usually goes up (more free water). However, the arrangement of water molecules in the active site is often different for different ligands. Docking software used in the present study did not offer modeling of explicit water molecules. Ligand interactions with water molecules were either neglected or taken into account using a mean-field approach as implemented in the scoring functions. Recent studies have shown a modest success in incorporating explicit water molecules in docking simulations [104,105,106]. Similar methodology applied to CYP3A4 may improve the accuracy of the DLR model in the future. Also, additional crystallographic studies on CYP3A4 ligands from the low-MW band may help in understanding the modalities of small ligand binding and expand the database of docking scores of the model.

**Figure 4:**
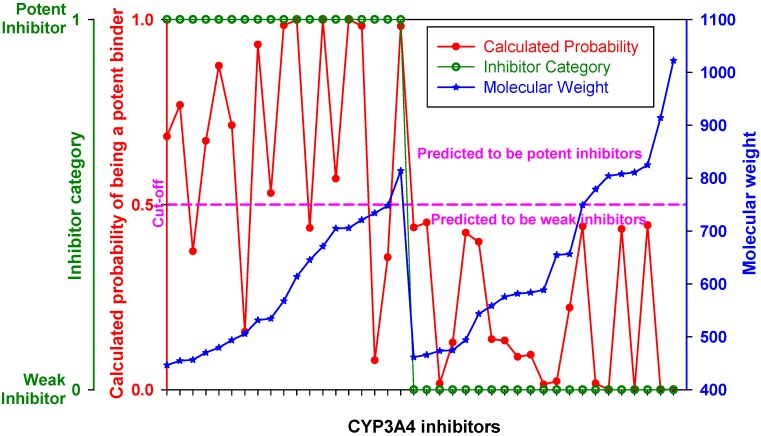
(**a**) ROC curve of the DLR model for the high-MW band (18 potent and 21 weak inhibitors). A, B, C, and D represent maximum specificity, maximum correct classification, equal sensitivity and specificity, and maximum sensitivity points, respectively. (**b**) Performance of the DLR model in the high-MW band.

Figure 4 represents the ROC curve and shows performance of the DLR model in the high-MW band of CYP3A4 inhibitors. The ROC AUC of the model using thus truncated applicability domain (TD-DLR) was 0.85, *i.e.*, the area outside the ROC curve shrunk by one quarter compared to the full-domain DLR model (FD-DLR). Similar to FD-DLR, the TD-DLR joint model had 100% specificity, while sensitivity and the rate-of-correct-classification improved to 72.2% (from 42.4%) and 87.1% (from 84.3%), respectively (Table 4). The average model rates-of-correct-classification for the split subsets (90% of the sample) and CV subsets (10% of the sample) of TD-DLR were 84.1% and 70.8%, respectively. That is, the cross-validated model rate-of-correct-classification for TD-DLR was almost 10% less than for FD-DLR. The latter, perhaps, was related to a smaller volume of training data in TD-DLR, which training set comprised less than one third of that of FD-DLR.

**Table 4:** Comparison of SDAR-, SAR- and DLR-modeling * methods.

Modelingmethod	Correctclassification			Sensitivity			Specificity			Type II error			Type I error			Numberof descriptors	
	Joint	Split	CV	Joint	Split	CV	Joint	Split	CV	Joint	Split	CV	Joint	Split	CV	Joint	Split/CV
SDAR	99.2	95.7	64.9	97.0	90.5	53.3	100	97.7	68.8	3.0	9.5	46.7	0	2.3	31.2	27	12 to 40
SAR	95.0	92.5	66.9	81.8	76.1	27.3	100	97.5	81.8	18.2	23.9	72.7	0	2.5	18.2	5	6.6
FD-DLR	84.3	83.8	79.3	42.4	44.1	35.5	100	98.7	95.7	57.6	55.9	64.5	0	1.3	4.3	7	7
TD-DLR	87.1	84.1	70.8	72.2	71.5	63.3	100	94.6	83.3	27.8	29.5	36.7	0	5.4	16.7	7	7
Consensus	95.0			81.8			100			18.2			0				

* The models reported are: “joint”, when all data of the training set was used in the model development (a single value is reported in the table), and “split”, when only 90% of the data were used in model development, while the remaining 10% were used for CV. The latter two sets of numbers are the averages over either 10 (DLR) or 100 (SDAR and SAR) random splits of the training set.

Only five drugs in the high-MW band were misclassified by TD-DLR. However, all of the drugs belonged to the category of potent inhibitors (as specificity of the model was 100%). The misclassified drugs were amiodarone, amprenavir, delavirdine, clarithomycin, and erythromycin.

**Figure 5:**
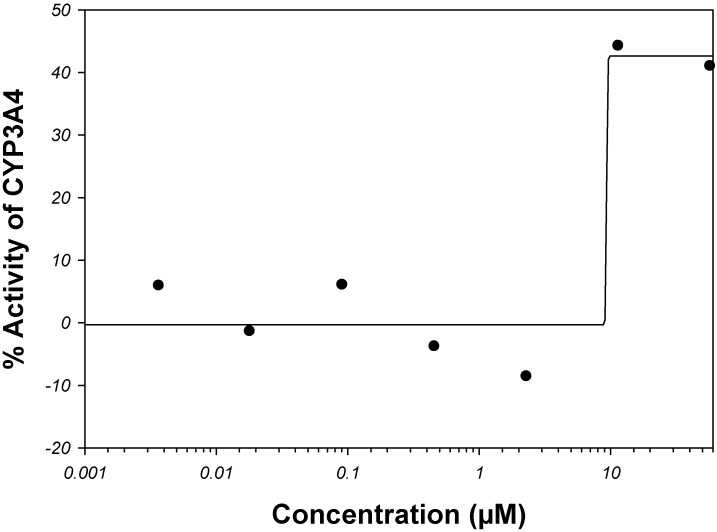
Dose-activity data of amiodarone as reported in PubChem^™^ bioassay AID 884 [68,107]. A four-parameter Hill equation was fitted to the data.

Amiodarone is a vasodilator. It is metabolized in the liver primarily by CYP3A4 and, at low concentrations, by CYP2C8 (see the Supplemental Material). The rate of catalysis of substrate luciferin-6'-phenylpiperazinylyl in the *in vitro* CYP3A4 system has increased in the presence of amiodarone, as given by PubChem^™^ bioassay AID 884 (Figure 5); therefore, amiodarone has been classified in PubChem^™^ as an activator and not inhibitor of CYP3A4 [68,107]. Although, potency of CYP3A4 inhibition may vary widely depending on a test substrate [57], we are unaware of examples in which the sign of reaction rate changes from the inhibition to activation. Of course, hypothetically, amiodarone can bind to an allosteric site outside the analyzed binding sites within the CYP3A4 active site pocket in such a way that it acts as an activator for the luciferin-6’-phenylpiperazinylyl substrate and an inhibitor for some other substrate(s). 

Amprenavir and delavirdine are the first-generation antiretroviral drugs for treatment of human immunodeficiency virus (HIV) infection. Amprenavir is a HIV protease inhibitor, while delavirdine is a non-nucleoside inhibitor of the HIV reverse transcriptase. Both amprenavir and delavirdine are mechanism-based inhibitors of CYP3A4. Presumably, delavirdine, binds covalently to the apoprotein and inactivates it; amprenavir is a substrate but its unknown reactive metabolites also inactivate the CYP3A4 isozyme [108,109,110]. Clarithromycin and erythromycin are macrolide ring antibiotics and also mechanism-based inactivators of CYP3A4. They are first metabolized by CYP3A4 to form reactive nitrosoalkanes via *N*-demethylation, which then impair the enzyme [108,109,110].

DLR is a mechanistic model. As such, it depends on and takes into account the potency of affine ligand binding of the parent compound. However, to exhibit potent clinical CYP3A4 inhibition effects, a mechanism-based inhibitor does not have to be a potent affine binder to CYP3A4, especially if the isozyme is inhibited by reaction metabolites but not the parent compound. If the parent compound does not have superior affine-binding properties, a high-fidelity DLR method shall not recognize it as a potent inhibitor.

In brief, one of the inhibitors (amiodarone) misclassified by the DLR model was, probably, also miscategorized in the literature, while all other misclassified compounds (amprenavir, clarithromycin, delavirdine, and erythromycin) were mechanism-based irreversible inactivators, to which the computational molecular docking approach does not necessarily apply. Taking into account that machine learning was conducted using clinical, not *in vitro*, data, it is surprising how reasonably well the DLR method performed on inhibitors from the high-MW band. It supports a conclusion of the previous section that only a small part of clinically-characterized CYP3A4 inhibitors disagrees with *in vitro* binding-assay data, while most of the clinically observed CYP3A4 inhibition effects originate from the potent affine binding of interfering agents directly at the active center of CYP3A4. Together with two other classifiers described in the following sections, the DLR model was incorporated in the consensus classifier, which was then applied to the EV set (see Section 2.6).

### 2.3. SDAR Classifier of Potent and Weak Inhibitors of CYP3A4

The average rate-of-correct-classification, sensitivity, and specificity of the SDAR discriminant analysis (DA) models were 95.7%, 90.5%, and 97.7%, respectively, during the training sessions with a randomly split dataset (retaining 90% of the data). The complementary 10% of the training set data were used for CV. During CV the average rate-of-correct-classification, sensitivity, and specificity were 64.9%, 53.3%, and 68.8%, respectively. Because each time the data selection was repeated (no information about independent variables was carried over from one run to another), CV for SDAR DA that is shown in Table 4 essentially was similar to external validation (EV). EV is also often called the “testing set” if the split of training data is conducted only once. In this sense, the rigor of the SDAR DA model was examined at a level deeper than conventional SAR/QSAR found in the literature (because the model never sees the EV until after the training is complete).

Rates of the type I and II errors for the SDAR DA model at the repeated-split training were only 2.3% and 9.5%, respectively. However, they increased to 31.2% and 46.7%, respectively, at conjugated EV. The low rates of false-positive and false-negative estimates at training and much higher rates at EV, perhaps, indicate that both the minor and major categories of inhibitors were under-represented by SDAR descriptors, *i.e.*, that the size of the training set was insufficient to extract a robust collection of spectral patterns representative of the minority and majority distributions. Although, the *F*-score was set to greater than four when selecting the spectral bins for the SDAR DA model, yet, overtraining of the classifier, perhaps, took place. Expansion of the training set is expected to stabilize the model.

The final model built using the full training set (referred as the “joint” model in Table 4) showed a 99.2% rate-of-correct-classification, 97.0% sensitivity, and 100% specificity. Only propofol, a potent inhibitor, was misclassified. Twenty-one of the 157 populated ^13^C-NMR bins and 6 of the 37 populated ^15^N-NMR bins were used during training of the joint SDAR DA model. A number of bins populated during the tenfold CV ranged from 12 to 40. Of the 27 bins in the joint SDAR DA model, several bins had higher coefficients. The ^13^C bin #176 was a major contributor to the class of potent inhibitors in the model. It was seen predominantly in the spectra of compounds with cyclic carbon atoms connected to the oxygen atom of a ketone group. Likewise, the ^13^C bin #193 was also important and had hits from two potent inhibitors, doxycycline and tetracycline. The ^13^C-NMR bin #47 was often present only in weak inhibitors (19 weak versus one potent inhibitor). The ^15^N bin #75 was present in five potent inhibitors, including four conazoles, and one weak inhibitor. The ^15^N bin #75 was found in inhibitors with heterocyclic five-member rings that contained more than one nitrogen atom, while the potent inhibitor ritonavir had a five-member heterocyclic ring with a nitrogen and sulfur atom. The SDAR, DLR, and SAR (see below) models were merged together by a consensus approach and then applied to the EV set as described in Section 2.6.

### 2.4. SAR Classifier of Potent and Weak Inhibitors of CYP3A4

The performance of the SAR classification model of potent and weak inhibitors of CYP3A4 was estimated by using a tenfold CV. Each cycle of the CV was repeated ten times by randomly splitting the training set into a training and testing subsets. In total, 100 decision forest (DF) classification models were generated and tested. At each iteration step the classifier was retrained, *i.e.*, no information was carried over from one step to another. CV carried out this way is at least as good as EV repeated ten times. The results showed an average split-training rate-of-correct-classification of 92.5% with a SD of 0.8%, and an average rate-of-correct-classification of 66.9% at testing with a SD of 3.3%. The results indicated that stable (low SD) classification models of potent and weak inhibitors of CYP3A4 can be constructed using the given training set. However, the prognostic power of the models was moderate. Perhaps, it was caused by chemical and structural diversity of compounds in the training set, for which accurate SAR/Mold^2^ classification models could be constructed but which could not be confidently extrapolated to the testing sets. With a larger training set, if coverage of the chemical space of CYP3A4 inhibitors becomes denser, the accuracy of cross-chemical extrapolation, perhaps, will increase.

**Figure 6:**
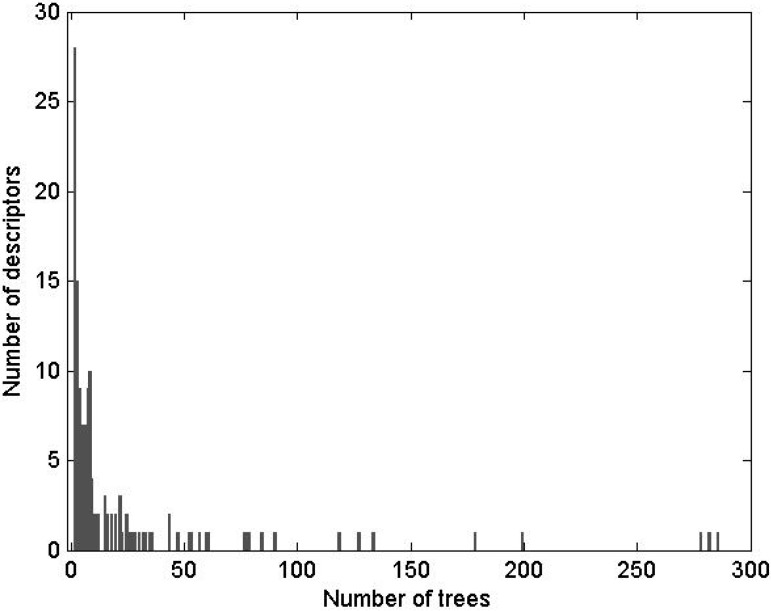
Distribution of molecular descriptors by DF decision trees built in the process of CV. Abscissa: the number of decision trees in which the same descriptor was used; ordinate: the number of descriptors that entered in the same number of decision trees.

The hypothesis about poor coverage of the chemical space was further evidenced by the analysis of the molecular descriptors used in the DF classification models. Each DF model had five decision trees; thus, a molecular descriptor could be used as many as 500 times (100 DF times 5 trees per DF model). Figure 6 depicts descriptors that were used in the classification models during tenfold CV. The x-axis indicates the number of trees in which a descriptor was used, while the y-axis shows the number of descriptors that were used by the same number of trees. A total of 145 molecular descriptors were used in the models. The average number of descriptors per tree was 6.6. Figure 6 shows that most of the descriptors were used by a small number of decision trees, indicating chemical and structural diversity among the different splits of the training set.

There were only three molecular descriptors that were used by more than half of the decision trees at CV. These top three contributors to the SAR DF models were the Balaban electronegativity weighted with Pauling-scale index, reciprocal Wiener-type maximum path index, and Balaban-type polarizability weighted index. These indices reveal that molecular shape (represented by the Wiener index), electronegativity, and polarizability are the most important quantities relevant to the potency of CYP3A4 inhibition. Most likely they express molecular properties of inhibitors that bind to CYP3A4, such as the activation energy and regioselectivity [111] and interactions of ionizable groups and permanent dipoles on CYP3A4 [112]. In this case, it would mean that the binding properties are essential for proper classification of potent and weak inhibitors, and that one of the categories in the training set, probably potent inhibitors, consists predominantly of CYP3A4 binders.

During CV, specificity of the SAR DF models was persistently greater than sensitivity. The specificity and sensitivity were 97.5% and 76.1% at split training, and 81.8% and 27.3% at testing, respectively (Table 4). The misbalance between specificity and sensitivity was caused perhaps by a disparity in the training set between positive (33 chemicals) and negative (88 chemicals) outcomes. Although, more fundamental reasons described above in the docking section could also take place, especially taking into account that the descriptors picked by SAR DF during training expressed more the mechanism of binding rather than other possible mechanism of CYP3A4 inhibition.

The final SAR model developed using the full training set showed 81.8% sensitivity and 100% specificity at the 95% rate-of-correct-classification of potent inhibitor classification (Table 4). Six potent inhibitors (amiodarone, clotrimazole, diltiazem, haloperidol, propofol, and sertraline) were misclassified. Only amiodarone had MW greater than 450 Da. Thus, the number of correctly classified potent inhibitors was 9, 18, and 27 for the low-MW and high-MW range, and the whole training set, respectively. All numbers were within the limits of statistical expectation described in the docking section.

### 2.5. Comparison of the DLR, SDAR, and SAR Classifiers

Three very different machine learning classifiers were developed in the present study, and each of them showed a fairly high rate-of-correct-classification. Table 4 shows a comparison of the DLR, SDAR, and SAR model performances during 9:1 split training and CV. The rate-of-correct-classification of the models during tenfold CV was 79.3% and 70.8% for FD- and TD-DLR, respectively, 64.9% for SDAR, and 66.9% for SAR. Although the methodologies of DLR, SDAR, and SAR model development were vastly different, the produced models shared a deep fundamental connection in terms of classification features that were employed by the models to describe the CYP3A4 inhibitors. The similarities embraced: (1) two major descriptors of the SAR model were based on electronegativity, while (2) the ^13^C and ^15^N-NMR spectra used in SDAR models were dependent on the electronegativity of the substituent groups connected to the atom; similarly, (3) most significant independent variables of the DLR model represented scores of the Hammerhead, FRED^™^, and Glide^™^ scoring functions. These scoring functions incorporate electronegativity either in the form of electrostatic potential, which determines the electronegativity, or its derivatives such as repulsive, polar, and salvation terms of the Hammerhead scoring function [113]. Because the DLR model essentially was a mechanistic binding model, the observed descriptor commonality suggested its association with the potency of CYP3A4 binding. In other words, all methods indicated that the category of potent inhibitors consisted mostly of potent CYP3A4 binders, while the category of weak inhibitors consisted either of predominantly weak binders or compounds that set off clinical manifestations of CYP3A4 inhibition by other mechanisms.

The question arises as to whether a potent clinically observed inhibitor of CYP3A4 must be its potent binder. An answer may reside with the sensitivity and specificity of the models. During CV the specificity was consistently higher than the sensitivity as given by all methods, and the final models came out as 100% specific (Table 4). This suggests that the probability of recognizing a weak inhibitor as potent was smaller than the probability of recognizing a potent inhibitor as weak. Since a pattern of potent inhibitors learned by the classifiers seemingly was associated with affine binding to CYP3A4, other mechanisms related to clinically devised potent inhibition probably were underrepresented in the training set and, *i.e*. potent inhibitors of CYP3A4 present in the training set that were not potent affine binders to CYP3A4 comprised a minor class. They were not appropriately learned by the classifiers because the size of training set was insufficient for that, and, therefore, they were misclassified by the models. This could be a likely reason for persistent inferior sensitivity observed for all models.

Similarly, all classification methods summarized in Table 4 showed a relatively high, 37–73% rate of false-negative estimates during CV. SAR and FD-DLR models had the highest rate of false-negative estimates. The false-negative rate was high for FD-DLR because the model identified in the training set only one of 15 potent inhibitors in the low-MW band. For the SDAR model, the true positive rate was low and the false-negative rate was high, perhaps, because the 1D NMR spectra did not contain enough chemical structure-specific information to adequately represent diversity of the training set in the chemical space. Inhibition of CYP3A4 is unlikely to originate from a single carbon or nitrogen atom as expressed by 1D NMR spectra, but rather from the entire chemical structural entity. The accuracy of SDAR models may improve as more structural information is added to the spectra, as has been shown for 3D-QSDAR models [114,115]. Supplementing the carbon and nitrogen chemical shifts with information about distances between the atoms provides an additional opportunity to more comprehensively describe the chemical properties of a compound and, thus, improve SDAR modeling.

Extrapolation from the classification results of the final joint models suggests that from 3% to 30% of non-affine inhibitors of CYP3A4 may be detected as potent in clinical studies. As discussed in the docking section, these non-affine inhibitors may be inducers, mechanism-based inhibitors, or perhaps agents intervening with the active transport and others. The suggested range of numbers is wider but similar to the 10% mismatch between the HTS measurements and results of clinical trials reported and discussed in Section 2.1.

Also, there could be compounds that were incorrectly assigned to a wrong category based on information that was available when the training set was compiled; propofol may be one of them. Initially, it was attributed to the category of potent CYP3A4 inhibitors based on information external to the Merck Manual (refer to the Supplemental Material). FD-DLR, SDAR, and SAR classified propofol as a weak inhibitor of CYP3A4 (while for TD-DLR it was outside the MW domain of the model). However, later after the work had been completed, propofol was categorized as a weak inhibitor of CYP3A4 in a new release of the Merck Manual [32].

SAR and TD-DLR both classified amiodarone (MW of 645 Da) as a weak CYP3A4 inhibitor. As summarized in the docking section, the mechanism of amiodarone interference with catabolism mediated by the CYP3A4 isozyme is at least more complex than trivial inhibition by affine binding. However, the final joint SDAR model classified amiodarone as a potent inhibitor. Taking into account that SDAR showed the lowest of all methods rate-of-correct-classification at CV, SDAR model over­training may be a reasonable explanation for the discrepancy.

As the SDAR model showed signs of overtraining, and applicability of the DLR model to the training set was restricted by MW, four additional compounds within the low-MW band shared false-negative classification from the final joint SAR model. These four compounds were clotrimazole, diltiazem, haloperidol, and sertraline. It remains to be seen whether these compounds were indeed misclassified by the SAR and FD-DLR models, or were they miscategorized during compilation of the training set (perhaps, due to erroneous or incomplete literature information). However, the following information was subsequently found in the literature:

(1) Clotrimazole is one of the most potent specific reversible inhibitors of CYP3A4 *in vitro*; it is even more potent than ketoconazole [116]. However, unlike ketoconazole, its clinical potency is uncertain [117], and its kinetics is unique *in vitro* [118], which suggests a uniqueness in the mechanism of its affinity to CYP3A4, perhaps, involving a distinctive binding modality different from the “mainstream” affine inhibitors;
(2) Diltiazem is an irreversible (mechanism-based) inactivator of CYP 3A4 [119];
(3) Haloperidol inhibition potency is substrate-specific, ranging from potent (with nifedipine as substrate) to weak (with testosterone) and less than weak (with felodipine or simvastatin) [120];
(4) Sertraline has been recently asserted as a weak inhibitor of CYP3A4 [121].

It appears that all low-MW drugs misclassified by both DLR and SAR show variability in terms of CYP3A4 inhibition, which singles out their unique machine learning patterns from the “major” pattern of potent inhibitors. Perhaps, these minor patterns were on the one hand, distinct enough from the “mainstream” to deserve separate clusters in the SAR analysis, but on the other hand, statistically underrepresented in the training set to form well-resolved-mode-of-action based clusters. Therefore, they were segregated by the SAR model in the group of false-negative estimates.

For compounds in the low-MW band, multiple ligand binding could be thought as a putative molecular-biologic reason for distinctions between the minor- and major-type learning patterns. At multiple ligand binding, some of the molecules may be actual inhibitor(s) of the enzymatic catalysis, while others may carry a role of homo- or hetero-effectors that change the molecular basis of the affinity patterns of the actual inhibitors. Apart from several examples of such “molecular symbiosis” (e.g., ketoconazole molecules in the active site of 2V0M [98]) little is known about the effectors. It is quite possible that the affinity patterns would be unique for each combination of multiple ligands. In this case, unlikely even a large database of CYP3A4 inhibitors would be representative of multiple discretized minority types that may be typical of CYP3A4 inhibitors in the low-MW band, which would mean the greatest challenge for both experimental (*in vitro* or clinical) and computational methods; the smaller is the molecular size (and the larger is the active site), the more likely misclassification and miscategorization will take place.

### 2.6. Development of a Consensus Classifier and Its Application to an External Set of Compounds

The developed classifiers were applied to an external testing set of 120 known inhibitors of CYP3A4 [122], which potency of inhibition has not been documented in the Merck Manual [32]. The DLR, SDAR, and SAR methods identified 25, 34, and 29 compounds, respectively, as potent inhibitors of CYP3A4. Presuming, both the training and testing sets were drawn from the same general population, and using proportion priors of the training set, the obtained numbers were within the expected CI range of 24–43. DLR, SDAR, and SAR identified of the testing set 9, 23, and 13 potent inhibitors among the 89 low-MW-band compounds, and 16, 11, and 16 potent inhibitors in the high-MW band (31 compounds), respectively (Table 5). Except for the low-MW-band-FD-DLR result, all other numbers were within the anticipated ranges of 10–25 and 9–20 for the low- and high-MW band, respectively.

For the FD-DLR model, the number of potent inhibitors in the low-MW band was slightly outside the calculated 95% confidence bounds on the mean. However, the direct one-tailed Z-test for two proportions, in which the *calculated* potent inhibitors in the low-MW band of the testing set (9/89) were gauged against the potent inhibitors *observed* in the low-MW band of the training set (15/82), suggested that the difference between the two proportion was insignificant at the 95% level (*p* > 0.0619), *i.e.*, FD-DLR performed as it is supposed to on the low-MW band of the testing set. Comparison of proportions of true positive outcomes of the FD-DLR model (*i.e.*, the calculated potent inhibitors) in the low-MW band using the testing and training sets produced a different result. The null hypothesis (9/89 = 1/82) was rejected at the 95% level (*p* < 6.65 × 10^−3^), and the alternative (9/89 > 1/82) was accepted. In the high-MW band, the test for proportions did not identify abnormalities in model performance using both the testing and training set data; the null hypothesis (16/31 = 13/39) was accepted at the 95% level (*p* > 0.0615). From these analyses it was concluded that FD-DLR did train appropriately on the training set, so that it could perform as expected in both the high- and low-MW bands, as suggested by the results of its application to the testing set. However, in the low-MW band of the training set it failed to identify a statistically significant number of potent inhibitors for an unknown reason, perhaps, because of a statistical fluctuation in the data.

**Table 5:** External-test compounds classified as potent inhibitors (P) of CYP3A4 by DLR, SDAR, and SAR in the low- (**a**) and high-MW (**b**) bands.

**(a)**	Compound *	**FD-DLR**	**SDAR**	**SAR**	**(b)**	Compound *	**TD-DLR**	**SDAR**	**SAR**
	**tioconazole**	P	P	P		**dalfopristin**	P	P	P
	corticosterone	P	P			**delapril**	P	P	P
	ditiocarb sodium	P	P			**gallopamil**	P	P	P
	econazole		P	P		**glipizide**	P	P	P
	oltipraz		P	P		astemizole	P		P
	piroxicam	P	P			calcium folinate	P	P	
	quercetin		P	P		dirithromycin	P	P	
	salbutamol	P	P			irinotecan	P		P
	trimethoprim		P	P		lopinavir	P		P
	troglitazone		P	P		midecamycin	P		P
	*almotriptan*		P			paclitaxel	P	P	
	*bifonazole*		P			raloxifene		P	P
	*carvedilol*		P			reserpine	P		P
	*dihydralazine*		P			*avasimibe*			P
	*dimethyl sulfoxide*	P				*barnidipine*		P	
	*disulfamide*			P		*benidipine*	P		
	*flutamide*		P			*buprenorphine*		P	
	*ipriflavone*		P			*cerivastatin*		P	
	*malathion*		P			*efonidipine*			P
	*mequitazine*		P			*flurithromycin*			P
	*mizolastine*	P				*josamycin*			P
	*nilvadipine*	P				*lercanidipine*			P
	*nimodipine*	P				*mibefradil*	P		
	*oxiconazole*			P		*roxithromycin*	P		
	*pantoprazole*			P		*terfenadine*			P
	*papaverine*			P		*vindesine*	P		
	*pioglitazone*			P		* Confidence in compound classification as potent inhibitor of CYP3A4 is denoted by bold, regular and *italics* font for probable, plausible, and uncertain consensus estimates, respectively. Probable and plausible estimates were taken as positive majority rules consensus outcomes for potent inhibition.			
	*prednisone*		P						
	*quinelorane*		P						
	*ranitidine*			P					
	*sertindole*		P						
	*sulpiride*		P						
	*theophylline*			P					
	*valdecoxib*		P						

A literature search suggested that of 14 low-MW inhibitors of the training set misclassified by DLR, four were mechanism-based inhibitors of CYP3A4 (cimetidine [123,124], diltiazem [119], isoniazid [121], and diclofenac [125]); six were, in fact, either relatively weak inhibitors or inhibitors of questionable potency (fluconazole [126,127,128,129], metronidazole [126,130,131], miconazole [129], propofol [32], sertraline [132], and voriconazole [133]); and for two of the inhibitors the experimental data were controversial (clotrimazole [117,118] and haloperidol [120]). The literature on norfloxacin and doxycycline was scant, which prevented interpretation of classification/categorization of these compounds. Nevertheless, even changing categorization of the six “relatively weak inhibitors or inhibitors of questionable potency” from potent to weak (*i.e.*, using 9/82 and 27/121 in hypothesis testing for the fraction of potent inhibitors in the low-MW band and on the whole domain, respectively) did not change any of the reported above results. Thus, no systematic reason in DLR misclassification of the low-MW compounds of the training set was identified, although the docking method, in general, is expected to perform better on larger compounds [134]. In this connection, the validity of classification of dimethyl sulfoxide (DMSO) as a potent inhibitor (Table 5a) was intriguing. DMSO (MW = 78 Da) was among the smallest compounds in both the training and testing sets. Surprisingly, DMSO has been reported as a strong inhibitor, which inhibits testosterone 6b-hydroxylation by CYP3A4 in a concentration-dependent manner [135]. On the other hand, the 1'-hydroxylation activity of midazolam, which is another standard laboratory substrate for CYP3A4, is only weakly inhibited by large amounts of DMSO [135]. DMSO was classified as a potent inhibitor only by DLR. Therefore, it seems that different classifiers partially learned from different aspects of the minority type populations of the training set. 

To increase confidence in classification, the classification methods developed in the present study were combined using a consensus approach. Several consensus strategies may apply to Boolean outcomes. They could be either of conjunction, disjunction, majority rules, percent agreement [136], or even artificial intelligence [60]. Conjunction and disjunction resulted, respectively, in five and 60 inhibitors of the testing set classified as potent. Both numbers were outside the expected CI range. The null hypotheses about proportions equality to the training set prior were rejected by the one-tailed Z-test test for two sample proportions at a more than 99.99% probability level.

Synthesis of results was carried out following the strategy of majority rules consensus. Using it, 23 inhibitors of the testing set were classified as potent (Table 5). Similar numbers for the low- and high-MW bands were 10 and 13, respectively. All the numbers were within the expectancy range. A hypothesis about identity of the fractions of estimated potent inhibitors in the low- and high-MW bands was rejected with a 99.99% probability in favor of the alternative that the high-MW range was enriched with potent inhibitors. This result, obtained by using the estimated potent inhibitors of the testing set, was similar to one reported above for the actual potent inhibitors of the training set. This circumstance suggests that the synthetic consensus model accurately learned information that was available in the training set, so that even a subtle statistical attribute was carefully reproduced.

A percent agreement consensus strategy was applied to assign confidence levels to the classification results. If potency of inhibitor classification was in full agreement among the DLR, SDAR, and SAR methods, classification of the inhibitor as potent was regarded as a ‘probable’ outcome. If the agreement was less than that but more than 50%, the outcome was deemed ‘plausible’. Estimates with below than 50% agreement between the methods were considered ‘uncertain’.

Dalfopristin, delapril, gallopamil, glipizide, and tioconazole were classified by the consensus model as probable potent inhibitors, and 18 other inhibitors were classified as plausible potent inhibitors of CYP3A4. Among the latter, seven chemicals were classified as potent inhibitors by both DLR and SDAR, five chemicals by DLR and SAR, and six chemicals by SAR and SDAR; *i.e.*, the frequencies of dual-model conjunction on the testing set of 120 compounds were similar.

A search revealed that about a third of the compounds attributed to the class of potent inhibitors have been described in the literature as potent inhibitors of CYP3A4. Two of the five probable potent inhibitors—dalfopristin, a Gram-positive antibiotic, and tioconazole, an antifungal imidazole—have been characterized in the literature as strong inhibitors of CYP3A4 [137,138]. Among the 18 plausible potent inhibitors of CYP3A4, potency information was found for lopinavir, oltipraz, quercetin, raloxifene, and troglitazone.

Lopinavir is a HIV reverse transcriptase inhibitor, *i.e.*, a drug from a class with many potent inhibitors of CYP3A4. It has also been reported as a strong inhibitor of CYP3A4 [32,139]. Quercetin is a plant flavanoid with anti-inflammatory properties. It has been described as a moderate-to-strong inhibitor of CYP3A4 [140]. Troglitazone belongs to the glitazone family of drugs, which are used to treat diabetes. Oltipraz and raloxifene are multiple action drugs, although both are often used as chemo-preventive anti-cancer agents. Troglitazone, oltipraz, and raloxifene form reactive metabolites (during CYP3A4 metabolism) that have been shown to covalently bind to CYP3A4 [141,142,143]. As mechanism-based inactivators, they may be considered potent inhibitors of CYP3A4. Interestingly, neither troglitazone nor oltipraz or raloxifene were classified as a potent inhibitor by DLR. These drugs corroborate a hypothesis that was formulated in the context of potent inhibitors of the training set misclassified by DLR (amprenavir, clarithromycin, delavirdine, and erythromycin). This hypothesis suggests that mechanism-based inactivators together with inhibitors, which affinity-based inhibition activity is confounded either by effector molecules or at a higher level of biochemical machinery (such as transcriptional induction), may be outside the domain of potent inhibitors recognized by DLR. So far, it appears that the docking method associates such inhibitors predominantly with weak potency of CYP3A4 inhibition.

Mechanism-based inhibitors, perhaps, can be modeled more accurately using inhibitor-structure-based methods, such as SDAR and SAR. Also, the LR part of the DLR model can be supplemented with inhibitor-structure-based QSAR descriptors, but that would be equivalent to using docking scores alongside structure-based descriptors in SDAR, SAR or other structure-activity methods. In any case, improvements in the machine classification of mechanism-based and other minority-type inhibitors would be possible only if they are sufficiently represented in the training set.

For the other 13 plausible potent inhibitors of CYP3A4 relevant information was not found. Thus, where experimental data were available, there was a fair correlation between the consensus estimates and experimental data. The proposed models may be useful in setting priorities for the experimental testing of drugs and chemicals for interactions with CYP3A4, for screening virtual libraries of compounds, and for interpreting HTS *in vitro* data and results of machine learning classifiers that rely on these data.

## 3. Experimental Section

### 3.1. Data Selection

The chemicals used for modeling in the present study were from a dataset of Yap and Chen [33,122]. These authors have compiled information on inhibitors and substrates of CYP isozymes from several literature sources that are used by clinicians. Inhibition properties of the chemicals have been cross-checked across several sources to ensure that interlaboratory variations in experimental protocols do not significantly affect the quality of the information in the dataset [33,122]. There were 241 CYP3A4 inhibitors in the Yap and Chen dataset, which were borrowed for the present study. The inhibition potency of these chemicals was attributed based on clinical categorization by the Merck Manual [32]. The compiled database contained potency information for more than the CYP3A4 isozyme, including 1A2, 2A6, 2B6, 2C8, 2C9, 2C19, 2D6, and 2E1 (see the Supplemental Material for details). Among them, 88 chemicals were identified as weak, 16 as moderate, and 17 as strong inhibitors of CYP3A4. Since the groups were grossly unequal, the strong and medium inhibitors were combined together to form a set of 33 ‘potent’ inhibitors. This way the training set of the models became more balanced, with a ratio of potent-to-weak inhibitors of 0.375. Table 6 and Table 7 list the training set of 121 potent and weak inhibitors of CYP3A4 that were used for training of the DLR, SDAR, and SAR models. The remaining 120 compounds (listed in the Supplemental Material) could not be categorized by their inhibition potency based on the information provided by the Merck Manual [34]. These 120 chemicals were used as a set for EV of the developed models. The inhibition potency of EV compounds was estimated using the three developed classification models and then the literature was searched to determine the accuracy of these estimates.

**Table 6:** Training set of potent (P) and weak (W) inhibitors of CYP3A4 in the high-MW band.

Compound	Molecular weight	Inhibitor category	Outcome bioassay-884	Average IC_50_ (µM) bioassay-884 *
verapamil	455	P		
delavirdine	457	P		
pimozide	462	W	Inactive	
cisapride	466	W		
nefazodone	470	P		
clofazimine	473	W	Inactive	
sildenafil	475	W	Active	10 ^†^
nicardipine	480	P		
glibenclamide	494	W	Active	5.99 ± 6.81 (3)
imatinib	494	P		
amprenavir	506	P		
ketoconazole	531	P	Active	0.13 ^†^
aprepitant	534	P		
doxorubicin	544	W		
atorvastatin	559	W		
nelfinavir	568	P		
zafirlukast	576	W		
ergotamine	582	W		
dihydroergotamine	584	W		
etoposide	589	W		
indinavir	614	P		
amiodarone	645	P	Inactive	
bromocriptine	655	W		
teniposide	657	W		
saquinavir	671	P		
atazanavir	705	P		
itraconazole	706	P		
ritonavir	721	P		
clarithromycin	748	P		
azithromycin	749	W	Inconclusive	
vinorelbine	779	W		
tacrolimus	804	W		
docetaxel	808	W		
vinblastine	811	W		
troleandomycin	814	P		
vincristine	825	W		
sirolimus	914	W		
quinupristin	1022	W		

* Number of repeated measurements (same compound provided by different manufactures) in the bioassay is shown in brackets and a 95% confidence; ^†^ an average standard error on the IC_50_ with multiple measurements was 6.90 µM.

**Table 7:** Training set of potent (P) and weak (W) inhibitors of CYP3A4 in the low-MW band.

Compound	Molecular weight	Inhibitor category	Outcome bioassay-884	Average IC50 (µM) bioassay-884 *
thiamazole	114	W	Inactive	
isoniazid	137	P	Active	9.92 ± 16.45 (2)
valproic acid	144	W	Inactive	
acetaminophen	151	W	Inconclusive	
hydralazine	160	W	Inconclusive	
chlorzoxazone	170	W	Inactive	
metronidazole	171	P	Inactive	
propofol	178	P	Inconclusive	
selegiline	187	W	Inactive	
dexmedetomidine	200	W	Active	0.16 ^†^
pilocarpine	208	W	Active	15.85 ^†^
methoxsalen	216	W	Active	10.50 ± 7.11 (3)
acetazolamide	222	W	Inactive	
lomustine	234	W		
phencyclidine	243	W		
cimetidine	252	P	Inconclusive	
primaquine	259	W		
cyclophosphamide	261	W		
ifosfamide	261	W	Inactive	
ticlopidine	264	W	Inconclusive	
mirtazapine	265	W		
nevirapine	266	W	Inactive	
orphenadrine	269	W		
venlafaxine	277	W	Inactive	
diazepam	285	W		
testosterone	288	W	Inactive	
anastrozole	293	W		
diclofenac	296	P		
cocaine	303	W		
entacapone	305	W		
fluconazole	306	P	Inconclusive	
sertraline	306	P		
fluoxetine	309	W		
methadone	309	W		
olanzapine	312	W	Inactive	
pergolide	314	W	Active	12.59 ^†^
efavirenz	316	W		
fluvoxamine	318	W		
norfloxacin	319	P	Inactive	
chloramphenicol	323	W	Inactive	
quinine	324	W		
midazolam	326	W		
clozapine	327	W	Inconclusive	
paroxetine	329	W		
ciprofloxacin	331	W	Inactive	
fentanyl	336	W		
danazol	337	W		
dextropropoxyphene	339	W		
methylprednisolone	339	W		
clemastine	344	W	Active	5.01 ± 8.47 (3)
clotrimazole	345	P	Active	0.07 ± 0.10 (3)
omeprazole	345	W	Active	10 ^†^
nifedipine	346	W	Active	11.29 ± 3.59 (2)
voriconazole	349	P		
oxybutynin	357	W		
rabeprazole	359	W		
nitrendipine	360	W		
prednisolone	360	W		
drospirenone	367	W		
lansoprazole	369	W	Active	20.48 ± 12.85 (2)
tamoxifen	372	W	Inconclusive	
haloperidol	376	P	Inactive	
mefloquine	378	W		
azelastine	382	W		
loratadine	383	W	Inactive	
felodipine	384	W		
nisoldipine	388	W		
betamethasone	392	W		
sulconazole	398	W		
amlodipine	409	W		
risperidone	410	W	Inactive	
fluvastatin	411	W		
ziprasidone	413	W		
diltiazem	415	P	Active	3.98 ^†^
miconazole	416	P		
losartan	423	W	Active	19.95 ^†^
pravastatin	424	W		
irbesartan	429	W	Active	15.85 ^†^
mifepristone	430	W		
doxycycline	444	P		
mitoxantrone	444	W	Active	25.12 ^†^
tetracycline	446	P		

*^,†^ The same as in Table 6.

### 3.2. Analysis of CYP3A4 Inhibition using HTS Bioassays

HTS bioassay outcomes were obtained from PubChem^™^ [28]. A query for CYP3A4 inhibition was performed, and 1,333 hits were found. However with few exceptions, all datasets contained less than a hundred chemicals. The exceptions were two HTS bioassays, AID 884 and 1851, both conducted by the National Institutes of Health Chemical Genomics Center. AID 1851 was a combined bioassay with inhibition data for five CYP isoforms, including CYP3A4 [67]. An IC_50_ has been reported for the chemical tested in the bioassay but the strength of their inhibition has not been categorized. AID 884 was a dedicated CYP3A4 inhibition assay, in which the inhibitors were categorized as active, inactive, and inconclusive [68].

Extrapolation from the *in vitro* data to *in vivo* inhibition activity was carried out following the FDA guidance [18]. The guidance is based on Michaelis-Menten kinetics for competitive inhibition. It can be shown that, under conditions of competitive inhibition, the apparent Michaelis constant of the substrate-to-product enzymatic conversion reaction, *K_m,i_*, is scaled from its uninhibited value *K_m_* by a factor of


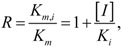
(1)

where [*I*] is the concentration of inhibitor and *K_i_* is the dissociation constant of the enzyme-inhibitor complex called the inhibition constant. At low concentrations of substrate, the Michaelis-Menten equation becomes


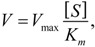
(2)

where *V* and *V*_max_ are the reaction velocity and maximum reaction velocity, respectively, and [*S*] is the concentration of substrate. When the substrate is bioconverted, *i.e.*, cleared, the reaction velocity can be expressed in terms of apparent intrinsic clearance, *CL*, as *V* = [*S*] × *CL*, and, therefore,


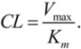
(3)

Since the dose or exposure expressed in terms of pharmacokinetic AUC is inversely proportional to the clearance, and the maximum velocity of reaction does not change under conditions of competitive inhibition,


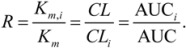
(4)

A concentration of inhibitor that decreases the rate of reaction at a specified concentration of substrate by half, IC_50_, is related to the inhibition constant by the equation of Cheng and Prusoff [144]:


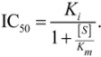
(5)

At low concentrations of substrate, *K_i_* ≈ IC_50_. Therefore,


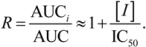
(6)

The equation implies competitive inhibition and that the substrate is metabolized by only one enzyme isoform in one metabolic pathway. The equation also holds for noncompetitive inhibition. Equation 6 becomes more complicated in case of uncompetitive inhibition or if several enzymes/pathways are involved.

Two approaches were exercised to estimate the concentration of inhibitor [*I*]: one by the MRDD and per oral bioavailability, and the other by the maximum concentration of inhibitor in serum (*C*_max_) that has been reported in clinical trials. The latter approximates the maximum hepatic inlet concentration. For the analysis presented in Table 1, a cutoff of 2 on *R* was applied for inhibition strength categorization. Drugs with *R* greater than 2 were categorized as P (potent, *i.e.*, a strong or moderate inhibitor), while those with *R* between 1.25 and 2 as W (weak inhibitor). This was in compliance with FDA guidance, in which inhibitors that increase the substrate AUC by fivefold or higher are labeled as ‘strong’, inhibitors that increase it between twofold and fivefold are labeled as ‘moderate’, and those that increase it between 1.25-fold and twofold are labeled as ‘weak’ [18].

### 3.3. Selection of CYP3A4 Crystal Structures for Docking

**Figure 7:**
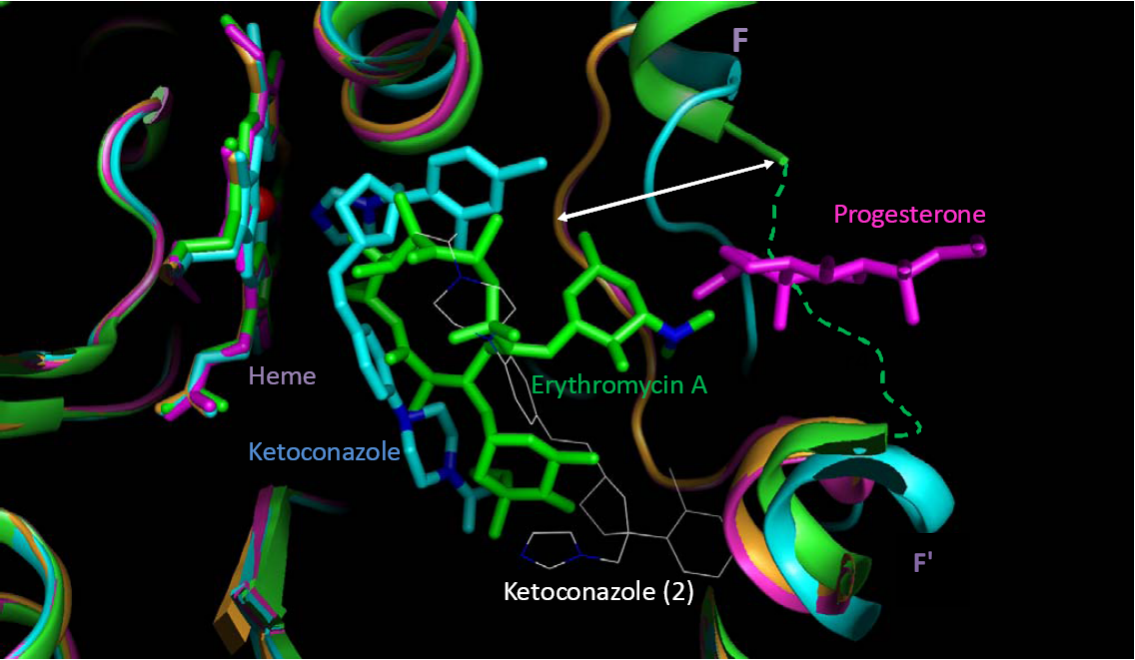
An active site view of superimposed crystal structures of the human CYP3A4 isozyme. 1TQN (no bound molecule) is shown in orange, 1W0F (with bound progesterone) in magenta, 2J0D (with bound erythromycin A) in green, and 2V0M (with bound ketoconazole) in cyan. The catalytic iron atom of the heme moiety is shown as a red van der Waals sphere. The active site volume is confined by the heme on the left and a flexible F–F′ loop that flanks the active site on the right. Nitrogenated sites-of-metabolism (SOM) on the ligands are shown in blue. As evidenced by the disposition of ketoconazole SOM in respect to the heme, ketoconazole is regioselectively bound at the substrate site next to the catalytic heme group (on the left), while erythromycin, although at the same site, is bound in a non-productive (inhibiting) orientation. Progesterone is bound at an allosteric binding pocket at the entrance of the active site of 1W0F (on the right). The active site of 1W0F is ligand-free and, thus, similar to ligand-free 1TQN as evidenced by overlapping positions of the flanking loop (left end of the white arrow). Upon substrate binding, the loop expands first to accommodate two ketoconazole molecules (2V0M, middle of the white arrow) and then further, to accommodate an even larger erythromycin A (2J0D, right end of the white arrow). Thus, the white arrow points at the most contracted and the most expanded conformations of the flanking F–F′ loop. The active site expands from the left to the right of the arrow to fit the size of substrate, and when the loop is at the right-most position (2J0D), volume of the active site is increased by more than 80% [98]. The green dashed trace denotes five disordered residues of the flanking loop, for which the coordinates have not been resolved in the 2J0D structure because of poor electron density in that region. Note the second ketoconazole molecule at the bottom (thin white); it is bound to the same crystal structure 2V0M but in a non-productive pose at an auxiliary pocket within the active site of CYP3A4, which expands to accommodate both molecules.

**Figure 8:**
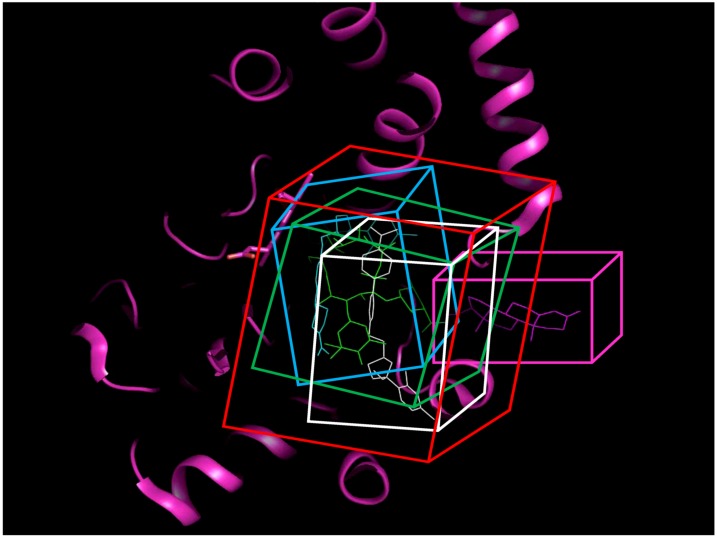
Superimposed docking sites of the human CYP3A4 isozyme. The trace of 1W0F structure (with bound progesterone) is denoted by the magenta ribbon. Each box represents a docking area of interest. The progesterone allosteric binding pocket area at the entrance of the 1W0F active site is represented by the magenta box; the catalytic site area of 2J0D (where erythromycin A is bound) is represented by the green box; the catalytic site area of 2V0M (where one of the ketoconazole molecules is bound) is represented by the cyan box; the auxiliary pocket area in the active site of 2V0M (that holds the second ketoconazole molecule) is represented by the white box; the area of the full active site in 2V0M is represented by the red box. The areas occupied by the catalytic site in 2V0M, 1W0F, and 1TQN structures are almost identical upon superposition. Therefore, the latter two are not shown for clarity.

Six crystal structures of human CYP3A4 isozyme were available from PDB [145] (codes: 1TQN, 2J0D, 1W0E, 1W0F, 1W0G, and 2V0M, 2.80 [98,146,147]). Their resolution and a free *R*-factor (*i.e.*, a statistics collected in crystallographic refinement using a EV set) ranged from 2.05 Å to 2.80 Å and from 0.271 to 0.318, respectively. Only the best quality structures among the structurally similar PDB codes were selected for docking studies. They included four structures, one without inhibitor (1TQN) and three with inhibitors (1W0F, 2J0D, and 2V0M) [98,146,147]. The heme group with a catalytic iron ion was well refined in all structures, as given by relatively low *B*-factors on the heme atoms. The substrate binding site was located next to the heme. A profound conformational change was observed in the structures with bound substrate/inhibitor. The conformational rearrangement involved opening of two loops flanking the active site to a different extent to accommodate the inhibitors of different sizes (Figure 7). Five residues in the loop connecting the F and F′ helices were absent in the 2J0D structure due to poor quality of the electron density in this region. Perhaps, the F-F′ loop in 2J0D was too flexible for adequate description by a single static set of mean coordinates, unlike the rest of the structure. CYP3A4 can metabolize various classes of substrates, and it does not follow the Michaelis-Menten type kinetics. Based on kinetic studies, Kenworthy’s group has proposed that three sub-pockets may exist within the active site of CYP3A4 [119]. Another hypothesis is that multiple kinetically distinguishable conformations of CYP3A4 are present, with or without substrate, effector, or inhibitor [14,146,148,149]. External factors such as interactions with CYP-reductase, cytochrome *b*_5_ and its reductase, and the presence of membrane phospholipids may stabilize particular conformations of CYP3A4 and affect its activity toward certain chemicals [146,150]. As shown in Figure 7 and Figure 8, the four selected structures represented different binding sites as well as different sizes/conformations of the CYP3A4 active site pocket, including expanded ones. 1TQN is a ligand-free structure with the smallest empty pocket [147]. A hydrophobic phenylalanine (Phe) cluster is located in proximity of the catalytic heme. A peripheral auxiliary binding site can be observed in the CYP3A4-progesterone complex (1W0F). It is located at the gate to protein surface, *i.e.*, it is distal to the heme and Phe cluster. It may be involved in the initial recognition of substrates or allosteric effectors [146]. The catalytic site of 1W0F was almost identical to 1TQN, 1W0G, and 1W0E. However, unlike these four structures, 2J0D and 2V0M revealed dramatic conformational changes upon ligand binding (Figure 7). In these two structures, the volume of active site was significantly increased (by more than 80% in 2J0D [98]). These two complexes represented two distinct expanded conformations of CYP3A4 because erythromycin A and ketoconazole induced different types of coordinate shifts in the isozyme.

In 2J0D, one erythromycin molecule is bound in a non-productive mode with the reactive D-desosamine group located 17 Å away from the heme iron atom [98]. However, in 2V0M a ketoconazole molecule resides within the bonding distance between its imidazole nitrogen atom and the iron atom of heme (referred to as first binding site in the docking procedure). Moreover, the active site of 2V0M enfolds the second ketoconazole molecule. It is stacked on the first one in an anti-parallel orientation [98] (referred as second binding site in the docking procedure), as shown in Figure 7. These structural features were consistent with wide substrate adaptability that is well-known for CYP3A4, suggesting that this isozyme possesses an extremely flexible and multifunctional active site pocket. Multiple binding modes and multiple binding sites on CYP3A4 were seen in the present study, which is consistent with atypical kinetics data for this isozyme [95,96,97].

### 3.4. DLR Methods

Docking studies were carried out using four programs: eHiTS^®^/CheVi^®^ (SimBioSys, Inc.; Toronto, Canada); FRED^™^ as a part of the OpenEye software suite (OpenEye Scientific Software, Inc.; Santa Fe, NM, USA); Surflex^™^ as implemented in Sybyl^®^ (Tripos, Inc.; St. Louis, MO, USA); and Glide^™^ as a part of the software suite from Schrödinger, Inc. (Portland, OR, USA).

No protein heavy atom minimization (or other resampling) was carried out in the present study. Coordinates of heavy atoms in modern crystallographic structures are already a product of geometry optimization consistent with experimental electron density. The electron density in turn is a time- and volume-averaged quantity. In the present work computational molecular docking was carried out using a PDB-deposited structure (rather than a thermodynamic ensemble of microscopic structural states in aqueous environment), thus, employing an unperturbed average structure was appropriate because it naturally represented the best averaging over a real physical-chemical thermodynamic ensemble observed in crystallographic experiments. The grand canonical ensemble was imitated by combining scores from four different crystallographic structures.

High chemical diversity of CYP3A4 substrates added complexity to docking modeling. Thus, for each of the four docking programs, seven docking sites were considered: 1TQN-catalytic site, 1W0F-catalytic site, 1W0F-entrance site, 2J0D-catalytic site, 2V0M-catalytic site, 2V0M-secondary binding site and 2V0M-full catalytic site. To avoid artifacts, the missing residues on the flanking loop of the 2J0D structure were left unreconstructed because molecular docking in this structure was focused at the catalytic site next to the heme and away from the unresolved part of the loop.

#### 3.4.1. eHiTS^®^

eHiTS^®^ implements a docking algorithm that involves a flexible ligand/rigid receptor methodology. It uses an exhaustive flexible-docking method starting with dividing the 3D structure of the ligand into rigid fragments and flexible chains. The rigid fragments are then docked independently into the receptor site and their poses, with flexible chains, are reconstructed by a graph-mating algorithm. The final poses are locally minimized and the score of each pose is reported.

3D coordinates for each the 121 training chemicals were generated using the Corina^™^ software (Molecular Networks, GmbH; Erlangen, Germany) as implemented by the Computer-Aided Drug Design Group at the National Cancer Institute [151]. Hydrogen atoms were added to each ligand. eHiTS^®^ automatically took into account all possible protonation states of each ligand–receptor pair, so no preliminary preparation of specific protonation states of ionizable ligands was necessary. Docking was performed by eHiTS^®^ Version 6.2 at the maximum accuracy setting. The docking area was set to 7 Å from the residues forming the active site. All other settings were kept at defaults. The score.sh script supplied with eHiTS^®^ Version 6.2 was used to calculate and rank the scores of docked compounds.

#### 3.4.2. FRED^™^

FRED^™^ relies on another exhaustive configurational searching algorithm. Unlike eHiTS^®^, instead of splitting ligand into fragments, it first generates all appropriate conformations of the ligand. Then it examines each pose within the protein active site, filtering them by shape complementarity and pharmacophoric features before selecting the final pose based upon a consensus of scoring functions.

Before actual docking, p*K*_a_s, protonation states and AM1-BCC partial charges were assigned to all atoms of the ligand using the Pkatyper^™^ and Molcharge^™^ programs from the QuACPAC^™^ 1.3.1 package. Then the Tautomers^™^ program from the same package was applied to enumerate possible tautomers. Tautomers at all possible levels were generated. The enantiomers and 3D coordinates of each compound were constructed by Omega^™^ 2.3.2 package. The default Omega^™^ settings were kept except for the following: the maximum number of output conformers (“maxconfs” option) was set to 500 and a root-mean-square Cartesian distance (“rms” option), below which two conformations were deemed duplicates, was set to 0.8 Å. Both QuACPAC^™^ and Omega^™^, along with FRED^™^, are parts of the OpenEye software suite. Receptor proteins were prepared using FRED-receptor^™^ 2.2.5. The shape of active site was described by two contours of the shape potential, an inner and outer one. The inner contour was set to 80–120 Å^3^, while the outer to 1,200–1,500 Å^3^. Docking was performed using FRED^™^ 2.2.3 in two steps, the shape fitting and optimization. During the shape-fitting step, a ligand was put into a 0.5-Å-resolution grid box around atoms of the active site using a smooth Gaussian potential. Several scoring functions were provided with FRED^™^, including Shapegauss, PLP, Chemgauss2, Chemgauss3, and CGO functions [152,153]. To rank the poses, a consensus scoring was used.

#### 3.4.4. Surflex^™^

3D structures of inhibitors from the training and test sets were generated using the Unity translator, which was a part of the local Sybyl^®^ 8.1 package. Subsequent docking was performed using the Surflex-Dock^™^ module. Surflex-Dock^™^ combines a scoring function from the Hammerhead docking system with a search engine that relies on a surface-based molecular similarity method [153,154,155]. Similar to eHiTS^®^, the ligand is fragmented into pieces before docking.

Each crystal structure was first preprocessed. Hydrogen atoms were added and minimized using the Structure Preparation Tool. The active site was identified and a conservative residue-based protomol was built with “proto_thresh” and “proto_bloat” set at 0.5 and 0.0, respectively. Then the protomol was visualized, its stray fragments were trimmed, and then the protomol was used to match the docking ligand fragments. Starting with the head fragment, other fragments were aligned one by one. A maximum limit of 50 conformations per fragment was set in each stage of the incremental construction process. Default settings were kept for all other parameters. Finally, the combined poses were refined and the 30 top-scored poses were reported.

#### 3.4.5. Glide^™^

The Glide^™^ algorithm aims for a complete systematic search of the conformational, orientational, and positional space of the docked ligand [156,157]. In this search, ligand conformations were first enumerated using LigPrep^™^, including the ionization states (a tool, Epik^™^, was used with target pH set at 7.0 ± 3.0), tautomers (as many as 16 tautomers per ligand), and enantiomers that specified which chiralities were retained; chiralities at other chiral centers were varied. The lowest energy ring conformation was produced for each inhibitor with a ring component. All enumerated structures were then filtered to remove structures that could cause subsequent processing failures either at the stage of energy minimization or at other stages, for instance, because of improper ionization state. After that, the geometries of retained structures were optimized.

The protein structure was pre-processed using the Protein Preparation Wizard. Bond order topologies were generated. Hydrogen atom positions were calculated and optimized for hydrogen bonding. For docking, Glide^™^ uses two boxes to define the active site: an outer grid-enclosing box and an inner ligand-diameter-midpoint box. The center of the outer box was determined by the centroid of the bound ligand as given by the original PDB file, or centroid of selected binding site residues if the ligand in the binding site was not present. Then the box was expanded to cover the whole active site. A 12-Å inner grid box was set to confine the ligand center. All other parameters were kept at their defaults. Docking precision was set at the extra precision (XP) level. During docking, the receptor was represented by the rigid grid, while the ligand was flexible. In the process of docking, an initial rough positioning and scoring was implemented to eliminate most unfavorable poses, which was followed by the grid energy optimization. Schrödinger GlideScore^™^ XP, a proprietary, empirical, multi-ligand scoring function, was applied. The geometry of poses of top-scored candidates was further optimized by full post-docking minimization, which relaxes strained ligand geometries and eliminates poses with unfavorable energy, *i.e.*, suboptimal bond length and angles, with eclipsing interactions, too many intraligand close contacts, and so on. The all-atom OPLS-2001 force field [158] was used. After that the poses were rescored using the GlideScore^™^ with a scaled OPLS term for non-bonded interactions. As many as 15 poses for each ligand were kept for reporting.

#### 3.4.6. Logistic Regression Modeling of Docking Scores and Statistical Analysis

LR models were built to combine the docking scores from 28 docking runs (four programs by seven binding sites in four CYP3A4 crystal structures). The docking scores were converted to standard scores (or Z-scores) before LR model construction. The SAS^®^ version 9.0.2 software was used (SAS Institute, Inc.; Cary, NC, USA). An automated stepwise regression procedure (forward and backward methods) [159,160] and manual selection of the best predictive variables were carried out. The selection of variables for the model was based on the Wald χ^2^ test. Variables, whose coefficients were statistically significantly different from zero with *p*-value of 0.05 or less, were recruited in the model. Note, when formation of the model is completed and the significance of coefficients is recalculated, the new and old *p*-values may not be the same. Models were evaluated based on their performance, number of parameters, and the ROC curves. Only models with ROC AUC greater than 0.8 were given further consideration. Therefore, in the manual selection procedure first variables, whose coefficients remained significant at α = 0.05 in the full model, were selected. Then, the manual model was supplemented with the least number (one) of additional variables with less-significant coefficients (*p*-value lesser than 0.1), which made model’s ROC AUC greater than 0.8. Chosen this way, the final manual LR model with the least number of parameters (seven) was adopted as a classifier of potent/weak CYP3A4 inhibitors, in which a LR probability cutoff of 0.5 was used to perform classification.

CIs for population proportions were calculated using Wilson’s method without continuity correction [161]. The identity of population proportions was tested using two-sample proportions procedures, as implemented in JMP^®^ 9.0.1 (SAS Institute, Inc.; Cary, NC, USA). SigmaPlot 10.0 (Systat Software Inc.; Chicago, IL, USA) was used for curve fitting.

### 3.5. SDAR Methods

SDAR modeling relates NMR spectra of a chemical to its activity [54], and SAR modeling relates substructural fragments, physical-chemical parameters of a chemical or both to its biological or toxicological activity or physical properties.

A *.mol file of each of the chemicals shown in Table 6 and Table 7 was processed using ACD/CNMR Predictor^™^ of the ACD/Labs^™^ version 12.0 software suite (Advanced Chemistry Development, Inc.; Toronto, Canada), its ^13^C-NMR spectrum calculated, and the spectrum exported in a text file. The ^15^N spectra for each compound were calculated using the ACD/NNMR Predictor^™^ software module from the same vendor. The ^13^C spectrum of each compound was binned by software written at NCTR using bin widths of 1 ppm (C1), while the ^15^N spectra were binned using the same software for bin widths of 5 ppm (N5). All discriminant analyses were performed using a Statistica^®^ version 8.0 software package (StatSoft, Inc.; Tulsa, OK, USA). DA training models of potent and weak inhibitors to CYP3A4 were built using C1 and N5 bins populated with at least three hits and using forward regression *F* > 4.0 to enter a bin to the DA model. No bins with only three hits were selected and only one bin had four hits. Previously, the forward regression on lower *F* was used to develop models for classification of inhibitors and non-inhibitors of CYP3A4 [122]. A more conservative SDAR DA model with smaller bin sizes was chosen for classifying potent and weak inhibitors in the present study, because there were expected to be fewer spectral and structural differences between potent and weak inhibitors than between inhibitor and non-inhibitors of CYP3A4. The SDAR classification DA models were constructed with prior probabilities of classifications (potent inhibitors/ weak inhibitors) set as equal for all DA SDAR models [162].

### 3.6. SAR Methods

Molecular descriptors are used to extract structural information in a form of numeric representation that is suitable for model development. Thus, the descriptors serve as the bridge between the molecular structure and biological activity of the chemical. Previously we developed Mold^2^, a software package for calculating 777 molecular descriptors [163]. Mold^2^ was used to calculate molecular descriptors for the training and test compounds used in the present work. Thereafter, Shannon entropy, also known as information entropy, was used to filter descriptors with low information content. This resulted in 327 of the descriptors remaining for modeling potent and weak inhibitors of CYP3A4. The resulting informative descriptors, with the assignment of compounds as potent or weak inhibitors of CYP3A4, were used to construct classification models.

DF [163,164,165], a classification method developed in our laboratories, is a novel pattern recognition method that combines the results of multiple distinct but comparable decision tree models to reach a consensus estimation. In training a DF model, five decision trees were generated by using Gini's diversity index for splitting the nodes in the trees. This process was used to construct classification models for estimating potent and weak inhibitors of CYP3A4 isozymes.

### 3.7. Cross-validation of SAR, SDAR, and DLR Models

Tenfold CV was used to measure the classification performance of SAR, SDAR, and docking models. At each iteration, the training data set was first randomly divided into ten equal portions, and then each was successively excluded from the training set and calculated using a model developed from the remaining nine portions. The rate-of-correct-classification at CV was taken as an average over ten iterations. Each random division of the data set into ten portions leads to ten specific pairs of training and test sets that could be biased in terms of the rate-of-correct-classification. The training and cross-validation models were evaluated in terms of their sensitivity, specificity, and the rate-of-correct-classification.

## 4. Conclusions

The CYP3A4 isozyme biotransforms a plethora of drugs and environmental xenobiotics. As such, it is instrumental in controlling the physiological levels of drugs and their metabolites. The mechanism for ligation to CYP3A4 can be either mediated by covalent bonding of the agent or its reactive metabolites, or by reversible binding of the inhibitor [166]. The binding of an inhibitor can occur at multiple sites on the isozyme, the structure of which appears to be very flexible [14,95,96,119,167]. In addition, modulation of the apparent catalytic activity *in vivo* may also occur at higher levels of biochemical organization [17,18]. Therefore, modeling potent and weak inhibitors of CYP3A4 can be challenging. Because of these potential complications, multiple independent modeling methods to classify potent and weak inhibitors of CYP3A4 were applied. Each of the models performed adequately, but not perfectly, as each method viewed the problem differently. The SDAR and SAR methods were primarily based on structure and physical descriptors that can be related to the chemical itself, while the DLR relied on scores of interaction energies between the chemical inhibitor and the enzyme. In this regard, the SDAR and SAR approaches are more general. Conceivably they can learn from different minority patterns embedded in the training set, provided that the training set is representative of each of them. In the present study, however, the size of the training set prevented realization of the full potential of SDAR and SAR methods, limiting their performance to largest uniform mode-of-action groups of inhibitors, which were either direct reversible binders or mechanism-based inactivators of the isozyme. In this situation, the DLR results appeared very favorable. Although, the DLR method hardly identified any mechanism-based inhibitor, it performed extremely well on reversible binders showing the highest overall results in CV. Taking into account the complexity of CYP3A4 and diversity of regulatory mechanisms, such an outcome was surprising. It confirmed a proposition that selective strong binders to the isozyme carry the greatest clinical importance. Also, it suggested that the large size of a reversible inhibitor is an important determinant of its high potency of inhibition. In this regard, comparison of clinical and HTS *in vitro* data was insightful. It indicated that in clinical trials, perhaps only a small fraction of modern drugs inhibits CYP3A4 activity by means other than direct interference with MFO. As the cost of *in vitro* screening decreases and study design improves, the HTS assessment of DDCIs may become a valuable adjunct in public health practice.

To increase confidence in machine classification, the DLR, SDAR, and SAR methods were combined by consensus modeling. When the consensus model was applied to an external testing set of 120 inhibitors, the consensus estimates were within the limits of statistical uncertainty (extrapolated from the testing set). Using a majority-rules consensus, five compounds of the testing set were classified as probable potent inhibitors (by all three methods). Subsequently, two of them were identified in the literature as known potent inhibitors. Eighteen additional compounds were classified as plausible potent inhibitors (by two of the three methods), and a literature search confirmed approximately a third of them. Likewise, when all of the models agreed that the chemical was a weak inhibitor of CYP3A4, no information was found in the literature stating that these chemicals are potent inhibitors. Many of these inhibitors may be partially responsible for changes in pharmacokinetics, pharmacodynamics, and possibly adverse reactions in people taking multiple drugs (polypharmacy), nutritional supplements, or exposed to hazardous chemicals. Therefore, these people should be either monitored by physicians or, at minimum, the chemical mixture, to which they are exposed to, be computationally evaluated for possible DDCIs before the polychemical therapy or exposure is started. The consensus methodology described in the present report provides means to do that. In the consensus, computational molecular docking supplied information that was complementary to traditional SAR and SDAR. Combined, these modeling methods provided a promising assessment of CYP3A4-mediated DDCIs, and suggested that a consonant use of multiple models may facilitate investigation of other complex systems in pharmaceutical and environmental toxicology.

## Supplementary Materials

Supplementary materials can be accessed at: http://www.mdpi.com/1420-3049/17/3/3407/s1.
