# Supplementary material for: Modeling Chemical Interaction Profiles: II. Molecular Docking, Spectral Data-Activity Relationship, and Structure-Activity Relationship Models for Potent and Weak Inhibitors of Cytochrome P450 CYP3A4 Isozyme
Source: Molecules. 2012 Mar 15;17(3):3407–60. doi: 10.3390/molecules17033407 (PMC6268819; doi:10.3390/molecules17033407)
Supplement: Supplementary file 1 [file molecules-17-03407-s001.pdf]

Supplementary Material

Modeling Chemical Interaction Profiles: II. Molecular Docking, Spectral Data-Activity Relationship, and Structure-Activity Relationship Models for Potent and Weak Inhibitors of Cytochrome P450 CYP3A4 Isozyme

Table 1. Testing set of CYP3A4 inhibitors (120 compounds).

Compound	Compound	Compound	Compound
Almotriptan	Domperidone	Paclitaxel	Tioconazole
Ambroxol	Ebrotidine	Pantoprazole	Trimethoprim
Amitriptyline	Econazole	Papaverine	Troglitazone
Aranidipine	Efonidipine	Parathion	Valdecoxib
Astemizole	Eplerenone	Perphenazine	Valspodar
Avasimibe	Ethinylestradiol	Pilsicainide	Vindesine
Azamulin	Etoricoxib	Pioglitazone	Zaltoprofen
Barnidipine	Famotidine	Piroxicam	Zileuton
Benidipine	Fluphenazine	Prednisone	Zolpidem
Bifonazole	Flurithromycin	Proadifen	
Buprenorphine	Flutamide	Quercetin	
Calcium folinate	Gallopamil	Quinelorane	
Carbamazepine	Gemfibrozil	Raloxifene	
Carvedilol	Gestodene	Ranitidine	
Cerivastatin	Glipizide	Reboxetine	
Cetirizine	Grepafloxacin	Remacemide	
Chlormadinone	Homochlorcyclizine	Reserpine	
Chloroquine	Ipriflavone	Rokitamycin	
Chlorpromazine	Irinotecan	Rosiglitazone	
Chlorpyrifos	Josamycin	Roxithromycin	
Ciclosporin	Lercanidipine	Salbutamol	
Clindamycin	Lilopristone	Seratrodast	
Colchicine	Lopinavir	Sertindole	
Corticosterone	m-Chlorophenylpiperazine	Simvastatin	
Cortisol	Malathion	Spironolactone	
Coumarin	Mequitazine	Stiripentol	
Dalfopristin	Metoclopramide	Sulfamethizole	
Daunorubicin	Metyrapone	Sulfamethoxazole	
Delapril	Mibefradil	Sulfaphenazole	
Desloratadine	Midecamycin	Sulfinpyrazone	
Dexamethasone	Mizolastine	Sulpiride	
Digoxin	Nilvadipine	Tazanolast	
Dihydralazine	Nimodipine	Tecastemizole	
Dimethyl sulfoxide	Norfluoxetine	Tegaserod	
Dirithromycin	Oltipraz	Terfenadine	
Disulfamide	Onapristone	Theophylline	
Ditiocarb sodium	Oxiconazole	Timoprazole	

Table 2. Categorization of the 241 CYP3A4 inhibitors of Yap and Chen [33] by potency of CYP isozyme inhibition.

Drug Name	Source	Inhibitor of CYP isoform-									Inhibitor of other protein	Group
		-1A2	-2A6	-2B6	-2C8	-2C9	-2C19	-2D6	-2E1	-3A4		
acetazolamide	Merck									weak		Training set
amiodarone	Merck	weak	moderate	weak		moderate	weak	moderate		moderate	P-glycoprotein	Training set
amlodipine	Merck	moderate	weak	weak	weak	weak		weak		weak		Training set
amprenavir	GreenRX pharmacy[1]						weak			strong		Training set
anastrozole	Merck	weak			weak	weak				weak		Training set
aprepitant	Merck					weak	weak			moderate		Training set
atazanavir	Merck	weak			weak	weak				strong	UGT1A1	Training set
atorvastatin	Merck									weak	P-glycoprotein	Training set
azelastine	Merck			weak		weak	weak	weak		weak		Training set
azithromycin	Merck									weak		Training set
betamethasone	Merck									weak		Training set
bromocriptine	Merck	weak								weak		Training set
chloramphenicol	Merck					weak				weak		Training set
chlorzoxazone	Merck								weak	weak		Training set
cimetidine	Merck	moderate				weak	moderate	moderate	weak	moderate		Training set
ciprofloxacin	Merck	strong								weak		Training set
cisapride	Merck							weak		weak		Training set
clarithromycin	Merck	weak								strong	P-glycoprotein	Training set
clemastine	Merck							weak		weak		Training set
clofazimine	AIDS journal[2]									weak		Training set
clotrimazole	Merck	weak	weak	weak	weak	weak	weak	weak	weak	moderate		Training set
clozapine	Merck	weak				weak	weak	moderate	weak	weak		Training set
cocaine	Merck							strong		weak		Training set
cyclophosphamide	Merck									weak		Training set
danazol	Merck									weak		Training set
delavirdine	Merck	weak				strong	strong	strong		strong		Training set

Drug Name	Source	Inhibitor of CYP isoform-									Inhibitor of other protein	Group
		-1A2	-2A6	-2B6	-2C8	-2C9	-2C19	-2D6	-2E1	-3A4		
dexmedetomidine	Merck	weak				weak		strong		weak		Training set
dextropropoxyphene	Merck					weak		weak		weak		Training set
diazepam	Merck						weak			weak		Training set
diclofenac	Medical report[3]	moderate			weak	weak			weak	strong		Training set
dihydroergotamine	Merck									weak		Training set
diltiazem	Merck					weak		weak		moderate		Training set
docetaxel	Merck									weak		Training set
doxorubicin	Merck			moderate				weak		weak		Training set
doxycycline	Merck									moderate		Training set
drospirenone	Merck	weak				weak	weak			weak		Training set
efavirenz	HIV medicine journal[4]					weak	weak			weak		Training set
entacapone	Merck	weak	weak			weak	weak	weak	weak	weak		Training set
ergotamine	Merck									weak		Training set
erythromycin	Merck	weak								moderate	P-glycoprotein	Training set
etoposide	Merck					weak				weak		Training set
felodipine	Merck				moderate	weak		weak		weak		Training set
fentanyl	Merck									weak		Training set
fluconazole	Merck	weak				strong	strong			moderate		Training set
fluoxetine	Merck	moderate		weak		weak	moderate	strong		weak		Training set
fluvastatin	Merck	weak			weak	moderate		weak		weak		Training set
fluvoxamine	Merck	strong		weak		weak	strong	weak		weak		Training set
glibenclamide	Merck				weak					weak		Training set
haloperidol	Merck							moderate		moderate		Training set
hydralazine	Merck									weak		Training set
ifosfamide	Merck									weak		Training set
imatinib	Merck					weak		moderate		strong	ABCG2	Training set
indinavir	Merck					weak	weak	weak		strong		Training set

**Drug Name**	**Source**	**Inhibitor of CYP isoform-**									**Inhibitor of other protein**	**Group**
		**-1A2**	**-2A6**	**-2B6**	**-2C8**	**-2C9**	**-2C19**	**-2D6**	**-2E1**	**-3A4**		
irbesartan	Merck				moderate	moderate		weak		weak		Training set
isoniazid	Merck	weak	moderate			weak	strong	moderate	moderate	strong		Training set
itraconazole	Merck									strong	P-glycoprotein	Training set
ketoconazole	Merck	strong	moderate	weak	weak	strong	moderate	moderate		strong	P-glycoprotein	Training set
lansoprazole	Merck					weak	moderate	weak		weak		Training set
lomustine	Merck							weak		weak		Training set
loratadine	Merck				weak		moderate	weak		weak		Training set
losartan	Merck	weak			moderate	moderate	weak			weak		Training set
mefloquine	Merck							weak		weak	P-glycoprotein	Training set
methadone	Merck							moderate		weak		Training set
methoxsalen	Merck	strong	strong			weak	weak	weak	weak	weak		Training set
methylprednisolone	Merck				weak					weak		Training set
metronidazole	Merck					weak				moderate		Training set
miconazole	Merck	moderate	strong	strong		strong	strong	strong	moderate	strong		Training set
midazolam	Merck				weak	weak				weak		Training set
mifepristone	Merck							weak		weak		Training set
mirtazapine	Merck	weak								weak		Training set
mitoxantrone	Merck									weak		Training set
nefazodone	Merck	weak		weak	weak			weak		strong		Training set
nelfinavir	Merck	weak		weak		weak	weak	weak		strong	P-glycoprotein	Training set
nevirapine	Merck	weak						weak		weak		Training set
nicardipine	Merck					strong	moderate	moderate		strong	P-glycoprotein	Training set
nifedipine	Merck	moderate				weak		weak		weak		Training set
nisoldipine	Merck	weak								weak		Training set
nitrendipine	Eur J Clin Pharmacol[5]									weak		Training set
norfloxacin	Merck	strong								moderate		Training set
olanzapine	Merck	weak				weak	weak	weak		weak		Training set
omeprazole	Merck	weak				moderate	moderate	weak		weak		Training set

Drug Name	Source	Inhibitor of CYP isoform-									Inhibitor of other protein	Group
		-1A2	-2A6	-2B6	-2C8	-2C9	-2C19	-2D6	-2E1	-3A4		
orphenadrine	Merck	weak	weak	weak		weak	weak	weak	weak	weak		Training set
oxybutynin	Merck				weak			weak		weak		Training set
paracetamol	Merck									weak		Training set
paroxetine	Merck	weak		moderate		weak	weak	strong		weak		Training set
pergolide	Merck*							strong		weak		Training set
phencyclidine	FDA website[6]									weak		Training set
pilocarpine	Merck		weak						weak	weak		Training set
pimozide	Merck						weak	weak	weak	weak		Training set
pravastatin	Merck					weak		weak		weak		Training set
prednisolone	Merck									weak		Training set
primaquine	Merck	strong						weak		weak		Training set
propofol	GreenRX pharmacy[7]	moderate			weak	weak	moderate	weak	weak	strong		Training set
quinine	Merck				moderate	moderate		moderate		weak		Training set
quinupristin	Merck									weak		Training set
rabeprazole	Merck				moderate		moderate	weak		weak		Training set
risperidone	Merck							weak		weak		Training set
ritonavir	Merck				strong	weak	weak	strong	weak	strong	P-glycoprotein	Training set
saquinavir	Merck					weak	weak	weak		moderate	P-glycoprotein	Training set
selegiline	Merck	weak	weak			weak	weak	weak	weak	weak		Training set
sertraline	Merck	weak		moderate	weak	weak	moderate	moderate		moderate		Training set
sildenafil	Merck	weak				weak	weak	weak	weak	weak		Training set
sirolimus	Merck									weak		Training set
sulconazole	Merck	weak	weak			weak	weak	weak	weak	weak		Training set
tacrolimus	Merck									weak	P-glycoprotein	Training set
tamoxifen	Merck			weak	moderate	weak				weak	p-glycoprotein	Training set
teniposide	Merck					weak				weak		Training set
testosterone	Merck									weak		Training set
tetracycline	Merck									moderate		Training set

Drug Name	Source	Inhibitor of CYP isoform-									Inhibitor of other protein	Group
		-1A2	-2A6	-2B6	-2C8	-2C9	-2C19	-2D6	-2E1	-3A4		
thiamazole	Merck	weak	weak	weak		weak	weak	moderate	weak	weak		Training set
ticlopidine	Merck	weak		moderate		weak	strong	moderate	weak	weak		Training set
troleandomycin	NASS document[8]									strong		Training set
valproic acid	Merck					weak	weak	weak		weak		Training set
venlafaxine	Merck			weak				weak		weak		Training set
verapamil	Merck	weak				weak		weak		moderate	P-glycoprotein	Training set
vinblastine	Merck							weak		weak		Training set
vincristine	Merck									weak		Training set
vinorelbine	Merck							weak		weak		Training set
voriconazole	Merck					moderate	weak			moderate		Training set
zafirlukast	Merck	weak			weak	moderate	weak	weak		weak		Training set
ziprasidone	Merck							weak		weak		Training set
Almotriptan	Merck											Test set
Amitriptyline	Merck	weak				weak	weak	weak	weak			Test set
Buprenorphine	Merck	weak	weak				weak	weak				Test set
Carbamazepine	Merck											Test set
Carvedilol	Merck										P-glycoprotein	Test set
Cetirizine	Merck											Test set
Chloroquine	Merck							moderate				Test set
Chlorpromazine	Merck							strong	weak			Test set
Colchicine	Merck											Test set
Cortisol	Merck											Test set
Daunorubicin	Merck											Test set
Desloratadine	Merck											Test set
Dexamethasone	Merck											Test set
Digoxin	Merck											Test set
Domperidone	Merck											Test set
Eplerenone	Merck											Test set

Drug Name	Source	Inhibitor of CYP isoform-									Inhibitor of other protein	Group
		-1A2	-2A6	-2B6	-2C8	-2C9	-2C19	-2D6	-2E1	-3A4		
Fluphenazine	Merck	weak				weak		weak	weak			Test set
Flutamide	Merck	weak										Test set
Gemfibrozil	Merck	moderate			strong	strong	strong					Test set
Glipizide	Merck											Test set
Irinotecan	Merck											Test set
Lopinavir	Merck							strong		strong	P-glycoprotein	Test set
Metoclopramide	Merck							weak				Test set
Metyrapone	Merck		weak									Test set
Nimodipine	Merck											Test set
Paclitaxel	Merck											Test set
Pantoprazole	Merck					weak	moderate				ABCG2	Test set
Perphenazine	Merck	weak						weak				Test set
Pioglitazone	Merck				moderate	weak	weak					Test set
Piroxicam	Merck					strong						Test set
Prednisone	Merck											Test set
Ranitidine	Merck	weak						weak				Test set
Reserpine	Merck										P-glycoprotein	Test set
Rosiglitazone	Merck				moderate	weak	weak					Test set
Simvastatin	Merck				weak	weak		weak				Test set
Sulfamethoxazole	Merck					moderate						Test set
Theophylline	Merck	weak										Test set
Tioconazole	Merck	weak	weak			weak	weak	weak	weak			Test set
Trimethoprim	Merck				moderate	moderate						Test set
Zileuton	Merck	moderate										Test set
Zolpidem	Merck											Test set

Drug Name	Source	Substrate of CYP isoform-										Substrate of other protein	Group
		-1A2	-2A6	-2B6	-2C8	-2C9	-2C18	-2C19	-2D6	-2E1	-3A4		
acetazolamide	Merck												Training set
amiodarone	Merck	minor			major at low conc.			minor	minor		major	P-glycoprotein	Training set
amlodipine	Merck										major		Training set
amprenavir	GreenRX pharmacy[1]				minor	minor					major		Training set
anastrozole	Merck												Training set
aprepitant	Merck	minor						minor			major		Training set
atazanavir	Merck										major		Training set
atorvastatin	Merck										major	P-glycoprotein	Training set
azelastine	Merck	minor						minor	minor		minor		Training set
azithromycin	Merck										minor		Training set
betamethasone	Merck												Training set
bromocriptine	Merck										major		Training set
chloramphenicol	Merck												Training set
chlorzoxazone	Merck	minor	minor						minor	major	minor		Training set
cimetidine	Merck											P-glycoprotein	Training set
ciprofloxacin	Merck											P-glycoprotein	Training set
cisapride	Merck	minor	minor	minor		minor		minor			major		Training set
clarithromycin	Merck										major		Training set
clemastine	Merck												Training set
clofazimine	AIDS journal[2]												Training set
clotrimazole	Merck												Training set
clozapine	Merck	major	minor			minor		minor	minor		minor		Training set
cocaine	Merck										major		Training set
cyclophosphamide	Merck		minor	major		minor		minor			minor		Training set
danazol	Merck												Training set
delavirdine	Merck								minor		major		Training set

Drug Name	Source	Substrate of CYP isoform-										Substrate of other protein	Group
		-1A2	-2A6	-2B6	-2C8	-2C9	-2C18	-2C19	-2D6	-2E1	-3A4		
dexmedetomidine	Merck		major										Training set
dextropropoxyphene	Merck										major		Training set
diazepam	Merck	minor		minor		minor		major			major		Training set
diclofenac	Merck	minor		minor	minor	minor		minor	minor		minor		Training set
dihydroergotamine	Merck										major		Training set
diltiazem	Merck					minor			minor		major	P-glycoprotein	Training set
docetaxel	Merck										major	P-glycoprotein	Training set
doxorubicin	Merck								major		major	P-glycoprotein	Training set
doxycycline	Merck												Training set
drospirenone	Merck										minor		Training set
efavirenz	Merck			major							major		Training set
entacapone	Merck												Training set
ergotamine	Merck										major		Training set
erythromycin	Merck			minor							major	P-glycoprotein	Training set
etoposide	Merck	minor								minor	major	P-glycoprotein	Training set
felodipine	Merck										major		Training set
fentanyl	Merck										major		Training set
fluconazole	Merck												Training set
fluoxetine	Merck	minor		minor		major		minor	major	minor	minor		Training set
fluvastatin	Merck				minor	major			minor		minor	SLCO1B1	Training set
fluvoxamine	Merck	major							major				Training set
glibenclamide	Merck					major					minor		Training set
haloperidol	Merck	minor							major		major		Training set
hydralazine	Merck												Training set
ifosfamide	Merck		major	minor	minor	minor		major			major		Training set
imatinib	Merck	minor				minor		minor	minor		major	P-glycoprotein	Training set
indinavir	Merck								minor		major	P-glycoprotein	Training set
irbesartan	Merck					minor							Training set
isoniazid	Merck									major			Training set

Drug Name	Source	Substrate of CYP isoform-										Substrate of other protein	Group
		-1A2	-2A6	-2B6	-2C8	-2C9	-2C18	-2C19	-2D6	-2E1	-3A4		
itraconazole	Merck										major		Training set
ketoconazole	Merck										major		Training set
lansoprazole	Merck					minor		major			major		Training set
lomustine	Merck								sub*				Training set
loratadine	Merck								minor		minor	P-glycoprotein	Training set
losartan	Merck					major					major		Training set
mefloquine	Merck										major		Training set
methadone	Merck			major		minor		minor	minor		major		Training set
methoxsalen	Merck		minor										Training set
methylprednisolone	Merck										major		Training set
metronidazole	Merck												Training set
miconazole	Merck										major		Training set
midazolam	Merck			minor							major		Training set
mifepristone	Merck										minor		Training set
mirtazapine	Merck	major				minor			major		major		Training set
mitoxantrone	Merck												Training set
nefazodone	Merck								major		major		Training set
nelfinavir	Merck					minor		major	minor		major	P-glycoprotein	Training set
nevirapine	Merck			minor					minor		major		Training set
nicardipine	Merck	minor				minor			minor	minor	major	P-glycoprotein	Training set
nifedipine	Merck								minor		major		Training set
nisoldipine	Merck										major		Training set
nitrendipine	Merck												Training set
norfloxacin	Merck												Training set
olanzapine	Merck	major							minor				Training set
omeprazole	Merck		minor			minor		major	minor		major		Training set
orphenadrine	Merck	minor		minor					minor		minor		Training set
oxybutynin	Merck										minor		Training set
paracetamol	Merck	minor	minor			minor			minor	minor	minor		Training set

Drug Name	Source	Substrate of CYP isoform-										Substrate of other protein	Group
		-1A2	-2A6	-2B6	-2C8	-2C9	-2C18	-2C19	-2D6	-2E1	-3A4		
paroxetine	Merck								major				Training set
pergolide	Merck*										major		Training set
phencyclidine	FDA website[6]												Training set
pilocarpine	Merck												Training set
pimozide	Merck	major									major		Training set
pravastatin	Merck										minor	P-glycoprotein, SLCO1B1	Training set
prednisolone	Merck										minor		Training set
primaquine	Merck										major		Training set
propofol	Merck	minor	minor	major		major		minor	minor	minor	minor		Training set
quinine	Merck	minor						minor			major		Training set
quinupristin	Merck												Training set
rabeprazole	Merck							major			major		Training set
risperidone	Merck								major		minor		Training set
ritonavir	Merck	minor		minor					major		major	P-glycoprotein	Training set
saquinavir	Merck								minor		major	P-glycoprotein	Training set
selegiline	Merck	minor	minor	major	minor			minor	minor		minor		Training set
sertraline	Merck			minor		minor		major	major		minor		Training set
sildenafil	Merck					minor					major		Training set
sirolimus	Merck										major	P-glycoprotein	Training set
sulconazole	Merck												Training set
tacrolimus	Merck										major	P-glycoprotein	Training set
tamoxifen	Merck		minor	minor		major			major	minor	major		Training set
teniposide	Merck										major	P-glycoprotein	Training set
testosterone	Merck			minor		minor		minor			minor		Training set
tetracycline	Merck										major		Training set
thiamazole	Merck												Training set
ticlopidine	Merck										major		Training set
troleandomycin	NASS												Training set

Drug Name	Source	Substrate of CYP isoform-										Substrate of other protein	Group
		-1A2	-2A6	-2B6	-2C8	-2C9	-2C18	-2C19	-2D6	-2E1	-3A4		
	document[8]												
valproic acid	Merck		minor	minor		minor		minor		minor			Training set
venlafaxine	Merck					minor		minor	major		major		Training set
verapamil	Merck	minor		minor		minor	minor			minor	major	P-glycoprotein	Training set
vinblastine	Merck								minor		major	P-glycoprotein	Training set
vincristine	Merck										major	P-glycoprotein	Training set
vinorelbine	Merck								minor		major		Training set
voriconazole	Merck					major		major			minor		Training set
zafirlukast	Merck					major							Training set
ziprasidone	Merck	minor									minor		Training set
Almotriptan	Merck								minor		minor		Test set
Amitriptyline	Merck	minor		minor		minor		minor	major		minor		Test set
Buprenorphine	Merck										major		Test set
Carbamazepine	Merck				minor						major		Test set
Carvedilol	Merck	minor				major			major	minor	minor	P-glycoprotein	Test set
Cetirizine	Merck										minor	P-glycoprotein	Test set
Chloroquine	Merck								major		major		Test set
Chlorpromazine	Merck	minor							major		minor		Test set
Colchicine	Merck										major	P-glycoprotein	Test set
Cortisol	Merck										minor	P-glycoprotein	Test set
Daunorubicin	Merck											P-glycoprotein	Test set
Desloratadine	Merck											P-glycoprotein	Test set
Dexamethasone	Merck										major	P-glycoprotein	Test set
Digoxin	Merck										minor	P-glycoprotein	Test set
Domperidone	Merck										minor		Test set
Eplerenone	Merck										major		Test set
Fluphenazine	Merck								major				Test set
Flutamide	Merck	major									major		Test set
Gemfibrozil	Merck										minor		Test set

Drug Name	Source	Substrate of CYP isoform-										Substrate of other protein	Group
		-1A2	-2A6	-2B6	-2C8	-2C9	-2C18	-2C19	-2D6	-2E1	-3A4		
Glipizide	Merck					major							Test set
Irinotecan	Merck			major							major	P-glycoprotein, SLCO1B1,UGT1A 1	Test set
Lopinavir	Merck										major		Test set
Metoclopramide	Merck	minor							minor				Test set
Metyrapone	Merck												Test set
Nimodipine	Merck										major		Test set
Paclitaxel	Merck				major						major	P-glycoprotein	Test set
Pantoprazole	Merck					minor		major	minor		minor		Test set
Perphenazine	Merck	minor				minor		minor	major		minor		Test set
Pioglitazone	Merck				major						minor		Test set
Piroxicam	Merck					minor							Test set
Prednisone	Merck										minor		Test set
Ranitidine	Merck	minor						minor	minor			P-glycoprotein	Test set
Reserpine	Merck												Test set
Rosiglitazone	Merck				major	minor							Test set
Simvastatin	Merck										major	SLCO1B1	Test set
Sulfamethoxazole	Merck					major					minor		Test set
Theophylline	Merck	major				minor			minor	major	major		Test set
Tioconazole	Merck												Test set
Trimethoprim	Merck					major					major		Test set
Zileuton	Merck	minor				minor					minor		Test set
Zolpidem	Merck	minor				minor		minor	minor		major		Test set

[1] http://greenrxpharmacy.com/drug_information_contents.aspx?PN=Amprenavir

[2] http://journals.lww.com/aidsonline/fulltext/2009/02200/pharmacology_of_second_line_antituberculosis_drugs.1.aspx

[3] http://www.mybwmc.org/library/41/041355

[4] http://www.hivmedicine.com/pdf/interact.pdf; Tseng A. www.tthhivclinic.com , General Hospital, Toronto, 2004.

[5] http://www.p.kanazawa-u.ac.jp/~taisha/ejcp2000_843.pdf

[6] http://www.fda.gov/drugs/developmentapprovalprocess/developmentresources/druginteractionslabeling/ucm093664.htm#classInhibit

[7] http://greenrxpharmacy.com/drug_information_contents.aspx?PN=Propofol

[8] http://www.spine.org/Documents/fentora_9-13-07.pdf

Note: Most of the data were collected from The Merck Manual for Healthcare Professionals (http://www.merckmanuals.com/professional/index.html) as provided by Lexi-comp (http://lexi-comp.com/). 79 out 120 testing-set compounds were not included in the table because the information was not available. Information in the database is subject to change because it is based on clinical data that are continuously refined and updated. This table represents a snapshot of the database as of 2010.
